# PR-DUB preserves Polycomb repression by preventing excessive accumulation of H2Aub1, an antagonist of chromatin compaction

**DOI:** 10.1101/gad.350014.122

**Published:** 2022-10-01

**Authors:** Jacques Bonnet, Iulia Boichenko, Reinhard Kalb, Mathilde Le Jeune, Svetlana Maltseva, Mattia Pieropan, Katja Finkl, Beat Fierz, Jürg Müller

**Affiliations:** 1 Laboratory of Chromatin Biology, Max-Planck Institute of Biochemistry, 82152 Martinsried, Germany;; 2 Laboratory of Biophysical Chemistry of Macromolecules, Institute of Chemical Sciences and Engineering (ISIC), Ecole Polytechnique Fédérale de Lausanne (EPFL), 1015 Lausanne, Switzerland

**Keywords:** Polycomb, PR-DUB, PRC1, H2AK119 monoubiquitination, Bap1, Asx, *Drosophila*

## Abstract

The Polycomb repressive complexes PRC1, PRC2, and PR-DUB repress target genes by modifying their chromatin. In *Drosophila*, PRC1 compacts chromatin and monoubiquitinates histone H2A at lysine 118 (H2Aub1), whereas PR-DUB is a major H2Aub1 deubiquitinase, but how H2Aub1 levels must be balanced for Polycomb repression remains unclear. We show that in early embryos, H2Aub1 is enriched at Polycomb target genes, where it facilitates H3K27me3 deposition by PRC2 to mark genes for repression. During subsequent stages of development, H2Aub1 becomes depleted from these genes and is no longer enriched when Polycomb maintains them repressed. Accordingly, Polycomb targets remain repressed in H2Aub1-deficient animals. In PR-DUB catalytic mutants, high levels of H2Aub1 accumulate at Polycomb target genes, and Polycomb repression breaks down. These high H2Aub1 levels do not diminish Polycomb protein complex binding or H3K27 trimethylation but increase DNA accessibility. We show that H2Aub1 interferes with nucleosome stacking and chromatin fiber folding in vitro. Consistent with this, Polycomb repression defects in PR-DUB mutants are exacerbated by reducing PRC1 chromatin compaction activity, but Polycomb repression is restored if PRC1 E3 ligase activity is removed. PR-DUB therefore acts as a rheostat that removes excessive H2Aub1 that, although deposited by PRC1, antagonizes PRC1-mediated chromatin compaction.

## Abstract (precis)

Here, Bonnet et al. investigated how levels of monoubiquitination of histone H2A at lysine 118 (H2Aub1) must be balanced for Polycomb repression, and show that in early embryos H2Aub1 is enriched at Polycomb target genes, where it facilitates H3K27me3 deposition by PRC2 to mark genes for repression. They show that PR-DUB acts as a rheostat that removes excessive H2Aub1 that, although deposited by PRC1, antagonizes PRC1-mediated chromatin compaction.

Polycomb group (PcG) proteins comprise a set of transcriptional regulators that control cell fate decisions in animals and plants. Most of the proteins classified as PcG regulators in *Drosophila* were originally identified because of homeotic phenotypes that are caused by the failure to repress HOX genes in cells where they ought to be inactive (Kassis et al. 2017).

The molecular and biochemical characterization of *Drosophila* PcG proteins revealed that they constitute the subunits of four types of protein assemblies: Polycomb repressive complex 1 (PRC1), Polycomb repressive complex 2 (PRC2), Pho repressive complex (PhoRC), and the Polycomb repressive deubiquitinase (PR-DUB) complex (Kassis et al. 2017). Among those, PRC2, PRC1, and PR-DUB are also conserved in mammals and possess enzymatic activity for modification of histone proteins in chromatin. PRC2 is a histone methyltransferase that monomethylates, dimethylates, and trimethylates histone H3 at lysine 27 (H3K27me1/2/3) (Laugesen et al. 2019; Yu et al. 2019). PRC1 contains the E3 ligase activity for monoubiquitination of histone H2A at lysine 119 in mammals (H2AK119ub1) and the corresponding lysine 118 in *Drosophila* (H2AK118ub1) that, for simplicity, we refer to here as H2Aub1 (Wang et al. 2004). Finally, PR-DUB functions as a deubiquitinase for H2Aub1 (Scheuermann et al. 2010). PRC1 also modifies chromatin through nonenzymatic mechanisms. First, PRC1 compacts nucleosome arrays and thereby renders them refractory to nucleosome remodeling (Francis et al. 2001, 2004; Grau et al. 2011), and second, PRC1 compacts chromatin by mediating long-range interactions between distant chromosomal sites (Isono et al. 2013; Kundu et al. 2017).

A main quest over the past two decades has been to understand the function of H3K27me3 and H2Aub1 in Polycomb repression. H3K27me3 acts as a binding site for the Polycomb (Pc) subunit of canonical PRC1 (Fischle et al. 2003; Min et al. 2003) and is also recognized by the Esc (EED) subunit of PRC2 (Laugesen et al. 2019; Yu et al. 2019). H3K27me3 is present in extended, block-like domains across PcG-repressed genes (Bracken et al. 2006; Schwartz et al. 2006; Bonnet et al. 2019), where it has a dual function. First, H3K27me3 marks these chromosome intervals for PRC1 binding to bring about gene repression through chromatin compaction. Second, PRC2 interaction with H3K27me3 forms a positive feedback loop that helps PRC2 to generate extended H3K27me3 domains (Laugesen et al. 2019; Yu et al. 2019). The demonstration that a K27R point mutation in histone H3 reproduces the phenotype of PRC2 mutants underscores the importance of H3K27me3 in Polycomb repression (Pengelly et al. 2013; McKay et al. 2015).

The function of H2Aub1, in contrast, has remained much more enigmatic (Barbour et al. 2020). H2Aub1 creates a binding site for the PRC2.2-specific accessory subunits Jarid2 and Aebp2 and thereby stimulates H3K27me3 deposition by this form of PRC2 (Kalb et al. 2014; Cooper et al. 2016; Kasinath et al. 2021). H2Aub1 also serves as binding site for Rybp (Arrigoni et al. 2006; Kalb et al. 2014; Zhao et al. 2020), a subunit that is specific to variant forms of PRC1 (vPRC1). vPRC1 complexes lack the H3K27me3 binding subunit Pc or the orthologous CBX2/4/6/7/8 proteins that characterize canonical PRC1 in flies and mammals, respectively (Gao et al. 2012; Tavares et al. 2012; Kang et al. 2022). The H2Aub1–Rybp interaction is thought to provide a positive feedback loop that enables vPRC1 complexes to efficiently build domains of H2Aub1-modified chromatin (Rose et al. 2016; Zhao et al. 2020).

In embryonic stem cells (ESCs) or epidermal progenitor cells in mice, H2Aub1 is enriched at Polycomb target genes in domains that coextend with H3K27me3 domains (Cohen et al. 2018; Fursova et al. 2019). Removal of PRC1 E3 ligase activity in ESCs results in widespread erosion of the genomic H3K27me3 pattern, supporting the view that the H2Aub1–PRC2.2 and H2Aub1–Rybp pathways make a major contribution to H3K27me3 domain formation (Endoh et al. 2012; Illingworth et al. 2015; Blackledge et al. 2020; Tamburri et al. 2020). However, even though H2Aub1 is required for viability in flies and mice, it is not an indispensable requisite for H3K27me3 domain formation and Polycomb repression (Illingworth et al. 2015; Pengelly et al. 2015; Cohen et al. 2018; Tsuboi et al. 2018). Unlike *Drosophila* that lack PRC2 catalytic activity, mutants lacking PRC1 E3 ligase activity or containing H2A with a mutated ubiquitination site fully maintain Polycomb repression at HOX genes (Pengelly et al. 2015). Moreover, chromatin compaction by PRC1 does not require H2Aub1 (Eskeland et al. 2010; Boyle et al. 2020). This is in contrast to the severe loss of Polycomb repression in flies carrying mutations in noncatalytic PRC1 subunits, which disrupt PRC1 chromatin compaction (King et al. 2005; Gambetta and Müller 2014). It has therefore been all the more puzzling that Polycomb repression is also defective in *Drosophila* mutants lacking PR-DUB catalytic activity for H2Aub1 deubiquitination (Scheuermann et al. 2010).

Here, we investigated the role of H2Aub1 and why its removal by PR-DUB is critical for Polycomb repression. We uncovered that in wild-type animals, H2Aub1 is subject to an unexpectedly dynamic regulation. H2Aub1 enrichment at Polycomb target genes in early embryos helps to establish H3K27me3 domains after zygotic genome activation. During subsequent development, PR-DUB then deubiquitinates H2Aub1 at Polycomb target genes to maintain them at a low level. This step safeguards Polycomb repression. We show that high density of H2Aub1 in nucleosome arrays prevents chromatin fiber folding in vitro, increases DNA accessibility at Polycomb target genes in vivo, and disrupts repression by PRC1 chromatin compaction.

## Results

### H2Aub1 is enriched at Polycomb target genes in early embryos but not during the stages when Polycomb keeps them repressed

**Figure 1.**
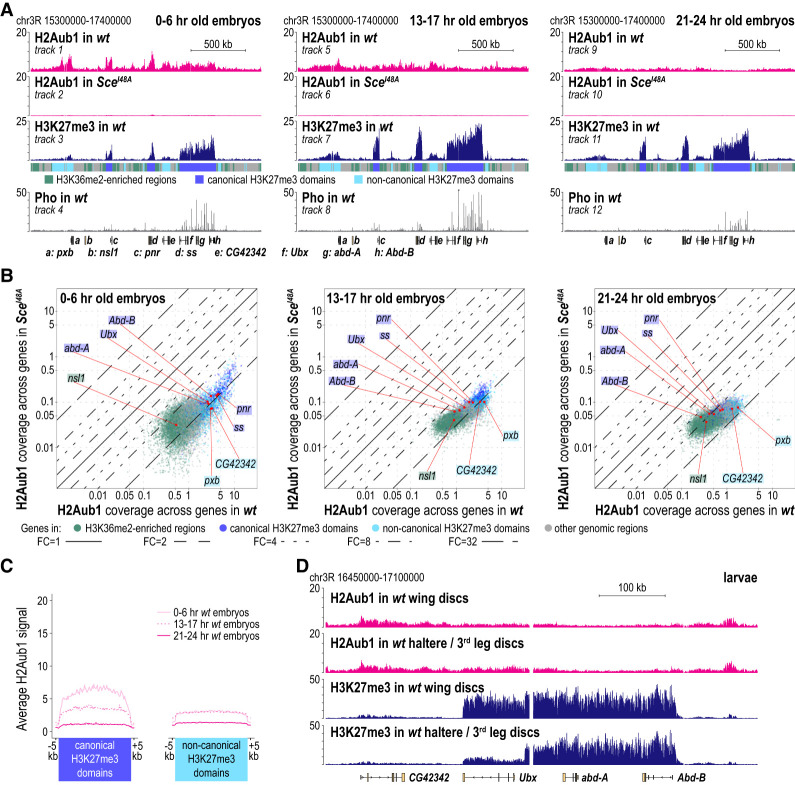
H2Aub1 is enriched at Polycomb target genes in early embryos but not during stages when Polycomb repression acts. (*A*) H2Aub1, H3K27me3, and Pho ChIP-seq profiles in 0- to 6-h-old, 13- to 17-h-old, and 21- to 24-h-old embryos of the indicated genotypes (Supplemental Table S1). Locations of selected genes in this chromosomal interval are indicated. (*B*) Scatter plots showing H2Aub1 read coverage across gene bodies in wild-type and *Sce*^*I48A*^ embryos at the same developmental time windows as in *A*. Each dot corresponds to a gene, and the color indicates the chromatin type in which the gene is located. Genes shown in *A* are marked as red dots, and the chromatin color code is indicated by the shading around the gene name. Lines indicating onefold, twofold, fourfold, eightfold, and 32-fold changes (FCs) are depicted. (*C*) Average distribution profiles of H2Aub1 at canonical and noncanonical H3K27me3 domains in early, midstage, and late stage wild-type embryos. (*D*) H2Aub1 and H3K27me3 ChIP-seq profiles in wing or haltere/third leg imaginal discs from wild-type third instar larvae.

We first analyzed the genome-wide distribution of H2Aub1 in early (0- to 6-h-old), midstage (13- to 17-h-old), and late stage (21- to 24-h-old) wild-type embryos. The use of spike-in chromatin for normalization allowed a quantitative comparison of the H2Aub1 ChIP-seq profiles between these different embryonic stages. As a control, we used *Sce*^*I48A*^ mutant embryos. The *Sce*^*I48A*^ mutation eliminates PRC1 E3 ligase activity but does not impair assembly of the complex (Buchwald et al. 2006; Pengelly et al. 2015). In the *Sce*^*I48A*^ mutant embryos used for ChIP-seq, both the zygotically expressed and maternally deposited Sce protein contained the Ile48Ala mutation (Pengelly et al. 2015). We previously found that H2Aub1 bulk levels are drastically diminished in such *Sce*^*I48A*^ mutant embryos (Pengelly et al. 2015). To relate the H2Aub1 profiles to the landscape of chromatin associated with Polycomb regulation, we also generated the profiles of Pho and of H3K27me3 in wild-type embryos. These profiles served as markers for what we refer to here as “canonical” and “noncanonical” H3K27me3 domains. Canonical H3K27me3 domains are characterized by high H3K27me3 enrichment and binding of Pho at Polycomb response elements (PREs); these regions therefore include the known Polycomb target genes (Supplemental Fig. S1A–C; Bonnet et al. 2019). Noncanonical H3K27me3 domains, in contrast, show only intermediate H3K27me3 levels and generally lack detectable binding of Pho or other PcG proteins (Supplemental Fig. S1A–C; Bonnet et al. 2019). Here, we color-coded chromosomal regions associated with canonical and noncanonical H3K27me3 domains in dark and light blue, respectively (Fig. 1A, colored bar below track 3). Chromosomal intervals that lack H3K27me3 enrichment and instead show enrichment of the active transcription mark H3K36me2 (see the Materials and Methods) are marked in green, whereas regions lacking enrichment of H3K27me3 or H3K36me2 are marked in gray (Fig. 1A, colored bar below track 3; Supplemental Fig. S1A).

Comparison of the H2Aub1 profiles in wild-type and *Sce*^*I48A*^ mutant embryos confirmed that the profiles in wild type indeed represent the distribution of H2Aub1-modified nucleosomes across the genome (Fig. 1A [cf. tracks 1, 5, and 9 with tracks 2, 6, and 10, respectively], B). Comparison between the different developmental stages in wild-type animals revealed that the H2Aub1 profile undergoes drastic changes during embryogenesis (Fig. 1A–C). In 0- to 6-h-old embryos, H2Aub1 is enriched in chromosomal regions that correspond to H3K27me3 domains (Fig. 1A, cf. tracks 1 and 3). In addition, there is also a low-level, near-uniform distribution of H2Aub1 across the entire genome (Fig. 1A, cf. tracks 1 and 3 at “green” and “gray” regions). In 13- to 17-h-old embryos, the H2Aub1 enrichment at H3K27me3 domains starts to decay; this is first notable at canonical H3K27me3 domains (Fig. 1A,B, left panel in C). In 21- to 24-h-old embryos, H2Aub1 is no longer enriched at either canonical or noncanonical H3K27me3 domains, and these regions all show the same low-level uniform H2Aub1 decoration as the rest of the genome (Fig. 1A [cf. tracks 1 and 9], B,C). In conclusion, even though H2Aub1 is enriched at Polycomb target genes in early embryos, the modification is largely depleted from these genes during the stages of embryogenesis when the Polycomb system acts to maintain repression at these genes (Struhl and Akam 1985; Simon et al. 1992; Soto et al. 1995).

To extend these analyses, we monitored H2Aub1 and H3K27me3 in wing imaginal discs and, in parallel, also in haltere and third leg discs from wild-type larvae. As reported previously (Papp and Müller 2006; Kang et al. 2017), in wing disc cells, a high-level H3K27me3 domain decorates *Ubx* and the adjacent *Bithorax complex* (*BX-C*) genes *abd-A* and *Abd-B*, all of which are repressed by Polycomb in these cells (Fig. 1D). In haltere and third leg disc cells, H3K27me3 was strongly reduced across the transcriptionally active *Ubx* gene, and the high-level H3K27me3 domain only covered *abd-A* and *Abd-B*, which are repressed in these cells (Fig. 1D; cf. Papp and Müller 2006; Kang et al. 2017). In contrast, only low levels of H2Aub1 were detected across *Ubx*, *abd-A*, and *Abd-B* both in wing and in haltere and third leg disc cells (Fig. 1D). H2Aub1 is therefore not enriched in the chromatin of canonical Polycomb target genes in cells in which they are repressed by Polycomb.

### PR-DUB antagonizes H2Aub1 accumulation at PRC1 target genes and shapes a low-level H2Aub1 landscape across the genome

To analyze the H2Aub1 genomic profile in embryos lacking PR-DUB catalytic activity, we generated embryos in which the catalytic Cys131 residue in the PR-DUB subunit Calypso (Caly; also called Bap1) was mutated to serine (Supplemental Fig. S2A). The Caly^C131S^ protein retains the ability to interact with its partner subunit, Asx, to form PR-DUB (Scheuermann et al. 2010), and its expression levels and nuclear localization are indistinguishable from that of the wild-type Caly protein (Supplemental Fig. S2B). For ChIP-seq, we used embryos in which both the zygotically expressed and the maternally deposited Calypso protein contained the Cys131Ser mutation (Supplemental Fig. S2C,D). H2Aub1 bulk levels in these *caly*^*C131S*^ mutant embryos were fourfold to fivefold higher than in wild type, recapitulating the increase in H2Aub1 levels in *Asx* mutant embryos (Supplemental Fig. S2E; cf. Scheuermann et al. 2010).

**Figure 2.**
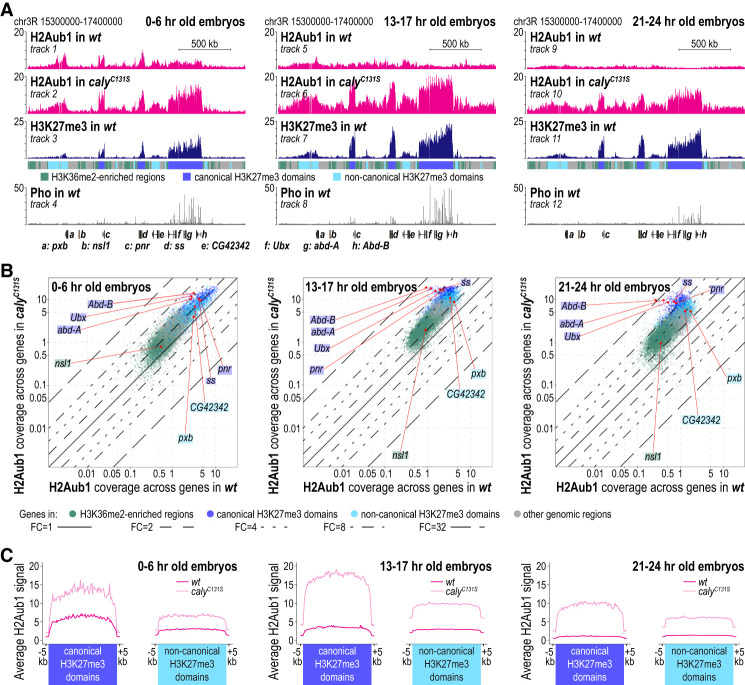
PR-DUB activity shapes a near-uniform, low-level H2Aub1 landscape during Polycomb repression. (*A*) H2Aub1, H3K27me3, and Pho ChIP-seq profiles in 0- to 6-h-old, 13- to 17-h-old, and 21- to 24-h-old embryos of the indicated genotypes (Supplemental Table S1). Genomic intervals are as in Figure 1A. (*B*) Scatter plots showing H2Aub1 read coverage across gene bodies in wild-type and *caly*^*C131S*^ embryos at the same developmental time windows as in *A*. Gene representation as colored dots and lines indicating fold changes are as in Figure 1B. (*C*) Average distribution profiles of H2Aub1 at canonical and noncanonical H3K27me3 domains in 0- to 6-h-old, 13- to 17-h-old, and 21- to 24-h-old wild-type and *caly*^*C131S*^ mutant embryos.

Comparison of the H2Aub1 profiles between *caly*^*C131S*^ mutant and wild-type embryos revealed that PR-DUB plays a central role in shaping the H2Aub1 landscape. In 0- to 6-h-old *caly*^*C131S*^ mutant embryos, the H2Aub1-enriched regions at canonical and noncanonical H3K27me3 domains showed, on average, a twofold increase in H2Aub1 levels, while the low-level genome-wide H2Aub1 levels were, on average, less than twofold higher than in wild-type animals (Fig. 2A–C). Intriguingly, at the *BX-C* genes, where H2Aub1 coverage in wild-type animals is lower than at most other Polycomb target genes, H2Aub1 levels in *caly*^*C131S*^ mutant animals were increased about fourfold compared with wild type (Fig. 2A,B). However, even more drastic changes in the H2Aub1 landscape were observed in 13- to 17-h-old and 21- to 24-h-old *caly*^*C131S*^ mutant embryos. First, the global H2Aub1 levels across the genome were, on average, about fourfold higher than in wild type (Fig. 2A,B). Second, at canonical H3K27me3 domains, H2Aub1 levels in *caly*^*C131S*^ mutants were more than fivefold higher than in wild type, and across the HOX genes *Ubx*, *abd-A*, and *Abd-B*, H2Aub1 levels were even 20-fold to 30-fold higher (Fig. 2A–C). This very high-level H2Aub1 accumulation precisely coextended with the canonical H3K27me3 domains at these regions (Fig. 2A [cf. tracks 6 and 7 with 10 and 11, respectively], C). The most straightforward interpretation of these findings is that, in wild-type animals, PRC1-type complexes extensively monoubiquitinate H2AK118 at Polycomb target genes also in late stage embryos but that H2Aub1 steady-state levels in these regions are normally kept low due to continuous deubiquitination by PR-DUB. It should be noted that not only PRC1 but also PR-DUB are bound at PREs of canonical Polycomb target genes (Scheuermann et al. 2010). In conclusion, the near-uniform H2Aub1 genome profile in late stage embryos appears to be generated by PRE-bound PR-DUB, which counteracts the high H2Aub1 deposition by PRC1 at canonical Polycomb target genes, and by untargeted PR-DUB, which acts genome-wide to reduce the low-level global H2Aub1 accumulation.

### H2Aub1 facilitates establishment of canonical H3K27me3 domains and is essential for formation of noncanonical H3K27me3 domains

To assess how H2Aub1 contributes to the formation of H3K27me3 domains, we compared the H3K27me3 profiles in wild-type, *Sce*^*I48A*^ mutant, and *caly*^*C131S*^ mutant embryos. The results from *Sce*^*I48A*^ and *caly*^*C131S*^ mutants are discussed in turn.

**Figure 3.**
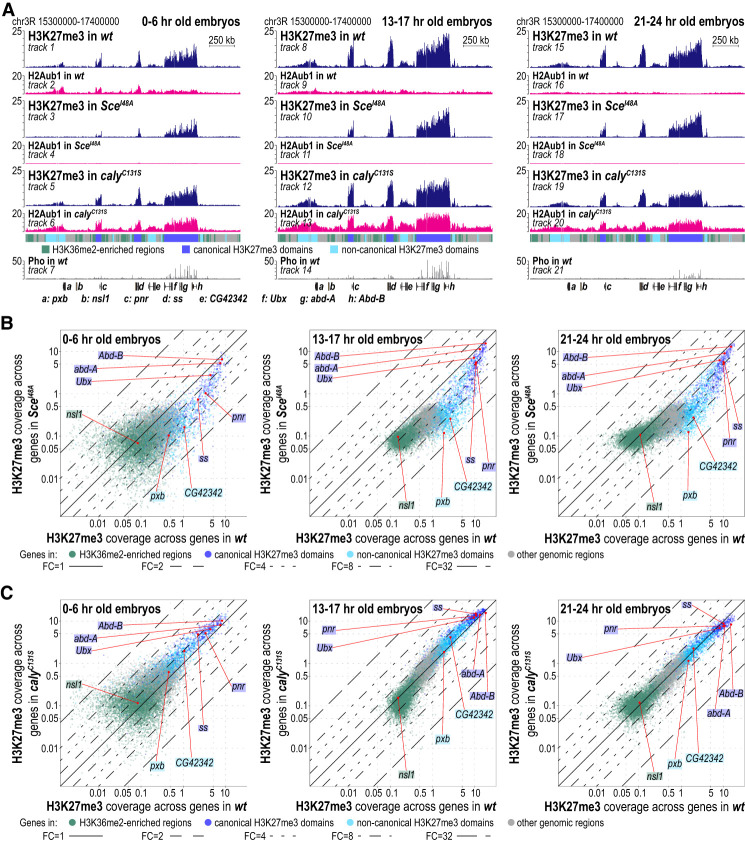
Formation of canonical and noncanonical H3K27me3 domains is differently affected in *Sce*^*I48A*^ mutants and largely unperturbed in *caly*^*C131S*^ mutants. (*A*) H2Aub1, H3K27me3, and Pho ChIP-seq profiles in 0- to 6-h-old, 13- to 17-h-old, and 21- to 24-h-old embryos of the indicated genotypes (Supplemental Table S1). Genomic intervals are as in Figure 1A. (*B*) Scatter plots showing H3K27me3 read coverage across gene bodies in wild-type and *Sce*^*I48A*^ embryos at the same developmental time windows as in *A*. Gene representation as colored dots and lines indicating fold changes are as in Figure 1B. (*C*) Scatter plots are as in *B* but comparing wild-type and *caly*^*C131S*^ mutant embryos.

In *Sce*^*I48A*^ mutant embryos, the absence of H2Aub1 affects canonical and noncanonical H3K27me3 domains differently. Canonical H3K27me3 domains are all formed in *Sce*^*I48A*^ mutants (Fig. 3A,B). However, the domains are established with slower kinetics than in wild type (Fig. 3A [cf. tracks 3 and 1], B). Moreover, H3K27me3 levels in the mature domains in *Sce*^*I48A*^ mutants are reduced in all instances, although to a variable extent and in most cases less than twofold (Fig. 3A [cf. tracks 10 and 17 with 8 and 15, respectively], B; Supplemental Fig. S3A). In contrast, at noncanonical H3K27me3 domains, H3K27me3 signals were reduced to background levels in *Sce*^*I48A*^ mutants (Fig. 3A,B; Supplemental Fig. S3A). In conclusion, this shows that H2Aub1 contributes to the establishment of canonical H3K27me3 domains at Polycomb target genes and is essential for formation of noncanonical H3K27me3 domains.

### Increased H2Aub1 levels in *caly*^*C131S*^ or *Asx*^*0*^ mutants allow formation of regular canonical and noncanonical H3K27me3 domains

To investigate whether the increase of H2Aub1 levels in PR-DUB mutants might alter H3K27me3 deposition across the genome, we analyzed the H3K27me3 profile in *caly*^*C131S*^ mutant embryos. The 0- to 6-h-old *caly*^*C131S*^ embryos showed a modest increase of H3K27me3 levels that appeared rather uniform across the entire genome (Fig. 3A,C; Supplemental Fig. S3B). On average, this increase in H3K27me3 coverage was less than twofold (Fig. 3C; Supplemental Fig. S3B). In 13- to 17-h-old and 21- to 24-h-old *caly*^*C131S*^ embryos, the H3K27me3 profile was largely comparable with that in wild-type embryos (Fig. 3C; Supplemental Fig. S3B). We emphasize that we found no genomic location where H3K27me3 levels in *caly*^*C131S*^ mutants were grossly diminished compared with wild type (Fig. 3C; Supplemental Fig. S3B).

To complement these observations, we also analyzed the H2Aub and H3K27me3 profiles in embryos lacking Asx. The H2Aub1 and H3K27me3 profiles in 21- to 24-h-old *Asx*^*0*^ mutant embryos closely resembled those in *caly*^*C131S*^ mutants. First, *Asx*^*0*^ mutants showed a fourfold increase in H2Aub1 levels genome-wide and an eightfold to 30-fold increase at canonical H3K27me3 domains compared with wild type (Supplemental Fig. S4A–C; cf. Fig. 2). Second, canonical and noncanonical H3K27me3 domains were formed like in wild-type animals (Supplemental Fig. S4A,D,E; cf. Fig. 3A,C and Supplemental Fig. S3B). We conclude that the lack of PR-DUB catalytic activity (i.e., *caly*^*C131S*^ mutants) or the complete removal of the complex (i.e., *Asx*^*0*^ mutants) (cf. Scheuermann et al. 2010) does not result in any gross changes in the H3K27me3 profile generated by PRC2.

### Elevated H2Aub1 levels disrupt Polycomb repression at HOX genes in a gene- and tissue-specific manner

In the following series of experiments, we used the phenotype of *Asx* and *caly* mutant animals as a functional readout to investigate how excess H2Aub1 disrupts Polycomb repression.

**Figure 4.**
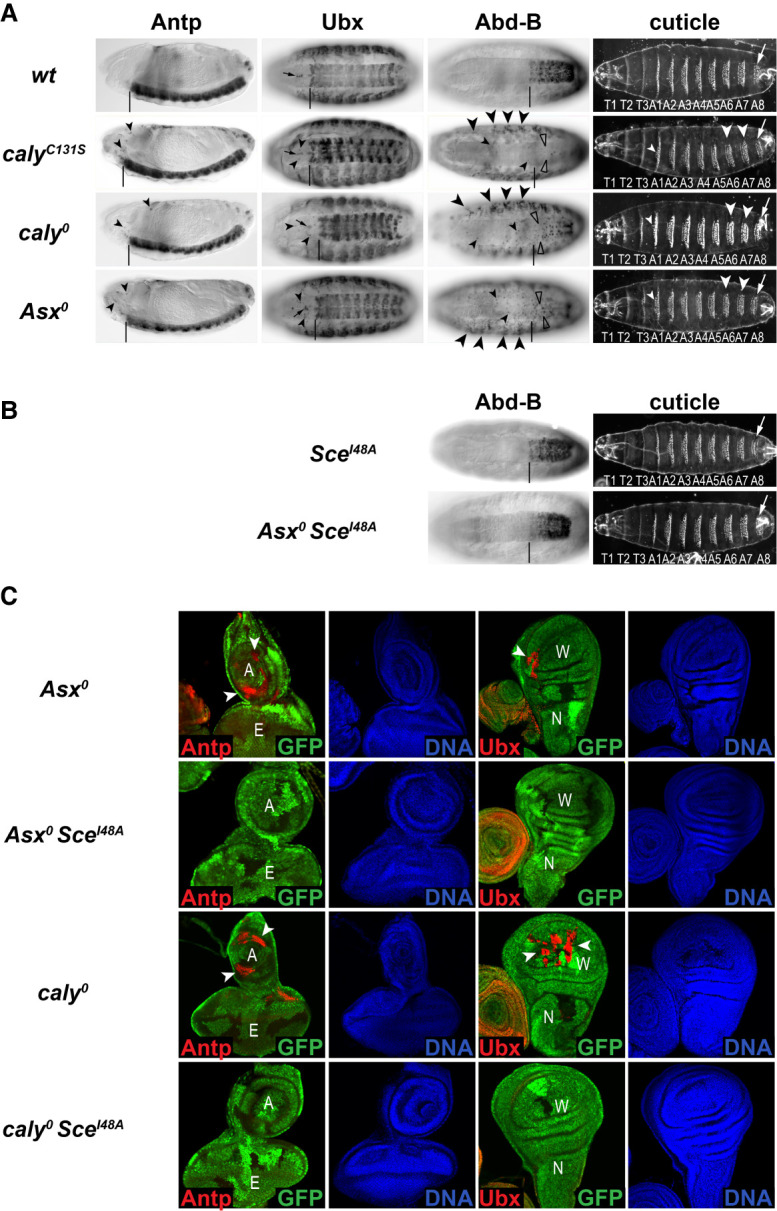
Excessive accumulation of H2Aub1 accounts for gene- and tissue-specific defects in Polycomb repression. (*A*) *caly*^*C131S*^, *caly*^*0*^, and *Asx*^*0*^ mutant embryos (Supplemental Table S1) show very similar defects in Polycomb repression. Side (first column) and ventral (second and third columns) views of stage 16 embryos, stained with antibodies against Antp, Ubx, or Abd-B as indicated, and embryonic cuticles (fourth column). Vertical lines mark the anterior boundaries of the Antp, Ubx, and Abd-B expression domains in wild type (wt); the small black arrows in the second column mark the four Ubx-positive midline cells that are part of the normal Ubx pattern in wt embryos. Note the stochastic misexpression of Antp, Ubx, and Abd-B in scattered individual cells in the CNS (small arrowheads point at some of those cells) in *caly*^*C131S*^, *caly*^*0*^, and *Asx*^*0*^ mutants; only Abd-B shows extensive misexpression in the epidermis (large arrowheads). In the CNS of all three mutants, there is loss of expression of Abd-B within its normal expression domain (empty arrowheads). The cuticles of all three mutants show comparable homeotic transformations of the A6 and A7 denticle belts (arrowheads) into denticle belts adopting the rectangular shape of the A8 denticle belt (arrow). In more anterior segments, the transformations are more subtle; e.g., the transformed first row of denticles in A1 (small arrowheads). In all three genotypes, these phenotypes were observed in 100% of the animals (*N > 30* for all genotypes). (*B*) Defective Polycomb repression in PR-DUB mutants is suppressed in animals that lack H2Aub1. (*Left*) Ventral views of stage 16 embryos that were *Sce*^*I48A*^ (*top*) or *Asx*^*0*^ *Sce*^*I48A*^ (*bottom*) (Supplemental Table S1), stained with antibody against Abd-B. (*Right*) Embryonic cuticles of the same genotypes. Abd-B expression and the embryonic cuticle of *Sce*^*I48A*^ mutant animals are indistinguishable from wild type (wt in *A*) (Pengelly et al. 2015). *Asx*^*0*^ *Sce*^*I48A*^ animals (26 out of 26 embryos) showed complete suppression of Abd-B misexpression (cf. *Asx*^*0*^ in *A*), and the cuticle of *Asx*^*0*^;*Sce*^*I48A*^ animals was indistinguishable from wt (in *A*). (*C*) Eye–antenna (first and second columns) and wing (third and fourth columns) imaginal discs with clones of *Asx*^*0*^ (first and second rows) or *caly*^*0*^ (third and fourth rows) mutant cells, induced in wild-type (first and third rows) or *Sce*^*I48A*^(second and fourth rows) homozygous mutant larvae (Supplemental Table S1). The eye (E), antenna (A), wing blade (W), and notum (N) primordia are indicated. In all cases, larvae were stained with antibodies against Antp or Ubx (red), as indicated, and with Hoechst to visualize DNA (blue); the *Asx*^*0*^ or *caly*^*0*^ homozygous mutant clone tissue is marked by the absence of GFP (green). In clones of *Asx*^*0*^ *Sce*^*I48A*^ or *caly*^*0*^ *Sce*^*I48A*^ double-mutant cells, Antp (second and fourth rows; *left*) and Ubx (second and fourth rows; *right*) remained completely repressed in all clones. The illustrated phenotypes were observed in 100% of cases (for each genotype, *N > 20* discs analyzed).

We first compared the embryonic phenotype of *caly*^*C131S*^ mutants with those of *Asx*^*0*^ and *caly*^*0*^ mutants. All three genotypes arrested development at the end of embryogenesis and showed homeotic transformations of posterior abdominal segments into the eight abdominal (A8) segment (Fig. 4A; cf. Breen and Duncan 1986; Gaytán De Ayala Alonso et al. 2007; Scheuermann et al. 2010). In the epidermis, the HOX gene *Abd-B* showed up-regulation within and misexpression anterior to its normal expression domain, whereas *Ubx* and *Antennapedia* (*Antp*) were misexpressed only in a few single cells anterior to their respective expression domains (Fig. 4A; cf. Simon et al. 1992; Sinclair et al. 1998; Scheuermann et al. 2010). In the central nervous system (CNS), *Abd-B* was deregulated in a more complex fashion, which is discussed elsewhere. Taken together, this shows that homeotic transformations are the main morphological defect not only in *caly*^*0*^ or *Asx*^*0*^ but also in *caly*^*C131S*^ mutant embryos. Moreover, misregulation of HOX genes is indeed caused by the lack of PR-DUB deubiquitinase activity. We also note that the extent of transcriptional deregulation of the different HOX genes in PR-DUB mutant embryos is highly gene- and tissue-specific and less widespread than in other PcG mutants. Removal of PR-DUB activity therefore does not globally disrupt Polycomb repression but impairs it in a gene-specific manner.

We reasoned that if defective HOX gene regulation in PR-DUB mutants is indeed caused by the increased H2Aub1 levels in these mutants, this deregulation should be suppressed in PR-DUB mutants in which H2Aub1 had been removed. This is indeed the case. *Asx*^*0*^ *Sce*^*I48A*^ double-homozygous embryos showed a normal *Abd-B* expression pattern, and their embryonic cuticle was indistinguishable from *Sce*^*I48A*^ single mutants or wild type (Fig. 4B). This strongly supports the conclusion that excess accumulation of H2Aub1 is the reason for deregulation of HOX gene expression in PR-DUB mutants.

To extend these findings, we also investigated the requirement for PR-DUB function during postembryonic development. We induced clones of *Asx*^*0*^, *caly*^*0*^, or *caly*^*C131S*^ homozygous mutant cells in heterozygous animals. In the first assay, we analyzed the morphology of differentiated epidermal structures formed by *Asx*^*0*^ or *caly*^*C131S*^ homozygous mutant cells in adult flies. Both *caly*^*C131S*^ and *Asx*^*0*^ mutants again showed highly similar phenotypes, with homeotic transformations as the only detectable morphological defects in epidermal tissues formed by the mutant cells (Supplemental Fig. S5A). These transformations only occurred in specific body regions, including the distal antennae, the wing blade, and the first tarsal segments of the legs (Supplemental Fig. S5A). We emphasize that adult structures formed by mutant cell clones in other regions of the body appeared morphologically normal (Supplemental Fig. S5A).

As expected from the locally confined homeotic transformations in adults, misexpression of HOX genes only occurred in specific areas of the imaginal disc primordia. Specifically, *Ubx* was widely misexpressed in *Asx*^*0*^, *caly*^*0*^, or *caly*^*C131S*^ mutant cell clones in the wing blade primordium of the wing imaginal disc but remained repressed in clones in the notum primordium (Fig. 4C; Supplemental Fig. S5A,B; cf. Gaytán De Ayala Alonso et al. 2007). Similarly, in the eye–antennal imaginal disc, *Antp* was mainly misexpressed in clones in the antenna primordium but remained repressed in most clones in the eye primordium (Fig. 4C; Supplemental Fig. S5A,B; cf. Halachmi et al. 2007). We conclude that removal of PR-DUB deubiquitinase activity at later stages of development also does not globally disrupt Polycomb repression at HOX genes but impairs it in a gene- and tissue-specific manner.

Analogous to the experiments in embryos, we also investigated how removal of PR-DUB affects HOX gene expression in larvae that lack H2Aub1. We induced clones of *Asx*^*0*^ or *caly*^*0*^ homozygous mutant cells in *Sce*^*I48A*^ zygotic mutant larvae. *Sce*^*I48A*^ zygotic mutants develop up to the pupal stage, and their larval tissues lack detectable amounts of H2Aub1 (Pengelly et al. 2015). Clones of *Asx*^*0*^ *Sce*^*I48A*^ or *caly*^*0*^ *Sce*^*I48A*^ double-mutant cells fully maintained repression of *Ubx* and *Antp*, like the neighboring *Sce*^*I48A*^ mutant tissue (Fig. 4C). These results corroborate that the failure to maintain Polycomb repression in PR-DUB mutant cells in larvae is indeed a consequence of increased H2Aub1 levels.

### The widely increased H2Aub1 levels in PR-DUB mutants do not cause global transcriptome changes

The remarkably specific homeotic phenotypes and absence of other obvious developmental abnormalities in PR-DUB mutants are perhaps surprising. To test for global transcriptome changes, we performed RNA-seq analyses on precisely staged single *Asx*^*0*^ mutant and wild-type control embryos (Supplemental Fig. S5C–F). The principal conclusion from these analyses is that the global increase in H2Aub1 levels across the genome in PR-DUB mutants does not cause global genome-wide deregulation of transcription. This is further corroborated by the observation that genome-wide RNA polymerase II occupancy is largely comparable between *Asx*^*0*^ mutant and wild-type embryos (Supplemental Fig. S5G,H).

### H2Aub1 deficiency does not globally disrupt Polycomb repression at developmental regulator genes

To complement these analyses, we also performed RNA-seq on *Sce*^*I48A*^ mutant embryos (Supplemental Fig. S5C–F), which revealed that there is no obvious deregulation of Polycomb target gene transcription in animals lacking H2Aub1. This is consistent with the observation that Polycomb repression at HOX genes is fully maintained in *Sce*^*I48A*^ mutants and that these animals do not show a Polycomb mutant phenotype (Fig. 4; cf. Pengelly et al. 2015).

### H2Aub1 inhibits chromatin fiber folding in vitro

**Figure 5.**
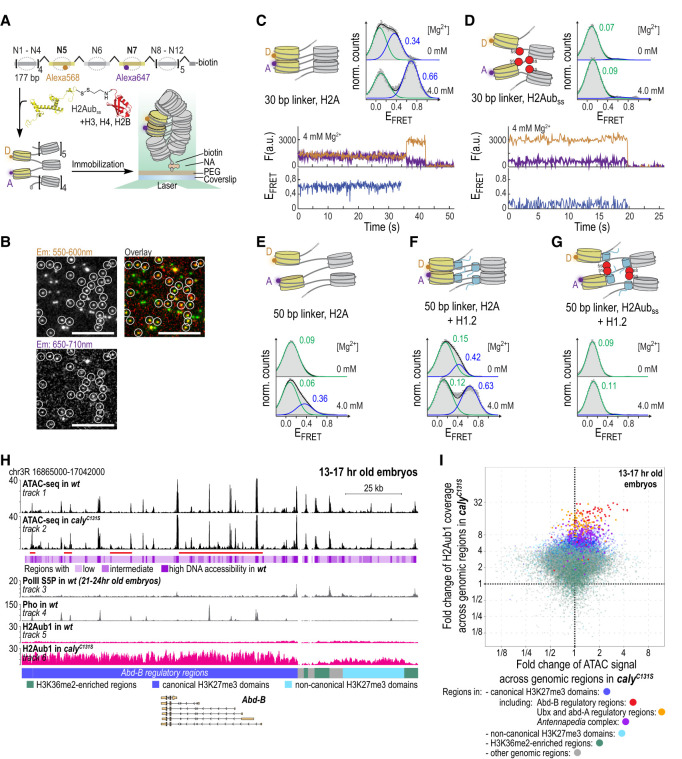
H2Aub1 perturbs nucleosome stacking and chromatin fiber folding in vitro and DNA accessibility in vivo. (*A*) Scheme of the smFRET experiment to detect nucleosome stacking in immobilized nucleosome arrays containing H2Aub_SS_, using TIRF microscopy. (*B*) TIRF microscopy images showing unmodified chromatin fibers at 4 mM Mg^2+^ under laser excitation at 532 nm at the indicated emission wavelengths or as an overlay showing FRET. White circles indicate the positions of immobilized fluorescently tagged chromatin. Scale bar, 10 µm. (*C*, *top*) FRET population histograms at the indicated Mg^2+^ concentrations. All histograms are the average of *n* > 2 independent repeats. Histograms are fitted with Gaussian functions (green and blue; black for the envelope). For the numbers of all repeats and for all fit values, see Supplemental Table S2. Peak E_FRET_ values for all populations are indicated. Error bars are standard error. (*Bottom*) Single-molecule traces (donor [orange], acceptor [violet], and FRET [blue]) for unmodified chromatin fibers using 30-bp linker DNA at 4 mM Mg^2+^. (*D*) smFRET traces and histograms for H2Aub_SS_-containing chromatin fibers with 30-bp linker DNA at the indicated Mg^2+^ concentrations. (*E–G*) smFRET histograms for unmodified chromatin fibers with 50-bp linker DNA (*E*), for unmodified chromatin fibers with 50-bp linker DNA saturated with H1.2 (*F*), and for H2Aub_SS_-containing chromatin fibers with 50-bp linker DNA saturated with H1.2 (*G*). (*H*) ATAC-seq profiles from 13- to 17-h-old wild-type (wt) or *caly*^*C131S*^ mutant embryos at the genomic interval containing *Abd-B*; for clarity, the locations of other transcription units in this region are not shown. RNA Pol II S5P, Pho, and H2Aub1 profiles are shown as a reference. Pho-bound PREs and RNA Pol II S5P-bound promoters show high DNA accessibility in both genotypes, but note the increased accessibility at *Abd-B* and its 3′ regulatory regions between peaks in *caly*^*C131S*^ mutants (red bar *below* track 2). Also note the high increase in H2Aub1 levels in *caly*^*C131S*^ mutants across this same region. (*I*) Scatter plots showing fold changes of ATAC-seq signal and H2Aub1 read coverage in 13- to 17-h-old *caly*^*C131S*^ mutant embryos. The dots represent genomic regions with high, intermediate, or low DNA accessibility and are colored according to the type of chromatin to which they map; all regions representing HOX gene sequences are labeled in color as indicated. See also Supplemental Figure S10B.

What is the mechanism of H2Aub1-mediated loss of repression in PR-DUB mutants? Previous studies had focused on the role of H2Aub1 as a protein binding platform (Kalb et al. 2014; Cooper et al. 2016; Kasinath et al. 2021). Ubiquitination at other histone residues has been shown to directly impact chromatin conformation (Fierz et al. 2011; Kilic et al. 2018a). We thus determined the structural impact of H2Aub1 on chromatin via a single-molecule fluorescence resonance energy transfer (smFRET) approach (Fig. 5A; Kilic et al. 2018b). For these experiments, we used human histones and chemically synthesized H2Aub1, where ubiquitin was connected to K119 via an asymmetric disulfide (H2Aub_SS_) (Fig. 5A; Supplemental Fig. S6; Chatterjee et al. 2010; Fierz et al. 2011; Debelouchina et al. 2017). H2Aub_SS_ is a close structural analog of H2Aub1, with the advantage of being chemically cleavable via reduction.

To detect chromatin compaction, we prepared a DNA template containing 12 copies of the 601-nucleosome positioning sequence separated by 30 bp of linker DNA. Moreover, we placed a FRET donor dye (Alexa fluor 568) and a FRET acceptor dye (Alexa fluor 647) within nucleosomes N5 (position *n)* and N7 (position *n+2)*, respectively (Supplemental Fig. S7), allowing detection of chromatin fiber folding via FRET (Fig. 5A; Kilic et al. 2018b). Using this DNA, we reconstituted chromatin fibers containing either unmodified H2A or H2Aub_SS_ (Supplemental Fig. S8). We then surface-immobilized these fibers in a flow cell, followed by imaging via total internal reflection fluorescence (TIRF) microscopy (Fig. 5A). From movies of donor and acceptor dye fluorescence emission under donor dye laser excitation (550 nm) (Fig. 5B), we calculated FRET efficiency (E_FRET_) time traces as well as time-averaged E_FRET_ histograms (Fig. 5B,C). Under low-salt conditions, unmodified chromatin adopted mostly an open state (E_FRET_ < 0.1), but a population of fibers exhibited medium FRET (E_FRET_ = 0.34), indicating transient nucleosome–nucleosome stacking interactions (Kilic et al. 2018b). Addition of bivalent cations (4 mM Mg^2+^) induced chromatin fiber folding, as indicated by a high FRET state (E_FRET_ = 0.66) (Fig. 5C). In contrast, we could not detect any FRET signal for chromatin containing H2Aub_SS_ independent of Mg^2+^, indicating a loss of nucleosome stacking (Fig. 5D). Importantly, deubiquitination of H2Aub_SS_ via reductive cleavage of the disulfide bond restored the ability of these chromatin fibers to fold (Supplemental Fig. S9).

The linker histone H1 is essential for the establishment of chromatin structure in metazoans. To test the impact of H1 on chromatin fiber folding, we assembled H2A- or H2Aub_SS_-containing chromatin fibers with an increased linker DNA length of 50 bp, observed in compact and inaccessible heterochromatin regions (Supplemental Fig. S7; Baldi et al. 2018). Interestingly, using unmodified H2A in the absence of H1, such chromatin fibers did not efficiently fold, presumably because of the increased internal flexibility imparted by the long linker DNA (Fig. 5E). However, upon incorporation of human linker histone H1.2 (Supplemental Fig. S8), fiber folding was restored (Fig. 5F). Conversely, the inclusion of H2Aub_SS_ completely antagonized the compacting effect of H1 (Fig. 5G), whereas the FRET signal could be restored after reductive deubiquitination of chromatin (Supplemental Fig. S9). Together, these findings demonstrate that H2Aub1 prevents stacking between *n* and *n+2* nucleosomes in nucleosome fibers and thus chromatin fiber folding, even in the presence of linker histones.

### Excessive H2Aub1 accumulation increases DNA accessibility at Polycomb target genes

We next tested whether in PR-DUB mutants the high density of H2Aub1 nucleosomes at HOX and other Polycomb target genes might also affect chromatin folding in vivo. To this end, we performed assays of transposase-accessible chromatin sequencing (ATAC-seq) to compare the accessibility of genomic DNA in *caly*^*C131S*^ mutant and wild-type embryos. We used genome segmentation (Supplemental Material) to define chromosomal intervals with high (violet), intermediate (magenta), and low (pink) accessibility in 13- to 17-h-old wild-type embryos (Fig. 5H, tracks 1 and 3). As expected, intervals with high accessibility included genomic sites such as gene promoters that are marked by RNA polymerase II occupancy or the nucleosome-depleted regions associated with PREs (Fig. 5H, cf. tracks 1, 3, and 4; Supplemental Fig. S10A, cf. tracks 1, 3, and 4) On the other hand, chromosomal regions with low accessibility would be expected to represent genomic intervals assembled into arrays of densely packed nucleosomes.

Comparison of the ATAC-seq profiles between *caly*^*C131S*^ mutant and wild-type embryos revealed that chromosomal regions with the highest increase in H2Aub1 coverage, including the HOX genes, show an overall increase in DNA accessibility in the mutant (Fig. 5I). The accessibility gain is particularly pronounced in genomic intervals in HOX genes that in wild-type animals fall into the class of low-accessibility regions (Supplemental Fig. S10B, right plot). The longest intervals in the genome with increased accessibility in *caly*^*C131S*^ mutants include regions of the *Abd-B* gene, including intervals 3′ to the *Abd-B* transcription unit (Fig. 5H; Supplemental Fig. S10C). Another example of a Polycomb target gene with a high increase in H2Aub1 levels and concomitant gain in DNA accessibility in *caly*^*C131S*^ mutants is illustrated in Supplemental Figure S10A.

In conclusion, these data show that at a subset of Polycomb target genes, the strong increase in H2Aub1 levels in PR-DUB mutants is associated with increased accessibility of the associated DNA. It remains to be determined whether the high density of H2Aub1 nucleosomes interferes with chromatin fiber folding at Polycomb target genes in the same way as observed in vitro or whether H2Aub1 increases DNA accessibility by, for example, reducing nucleosome occupancy.

### Increased H2Aub1 levels does not impair PcG protein complex binding to PREs

**Figure 6.**
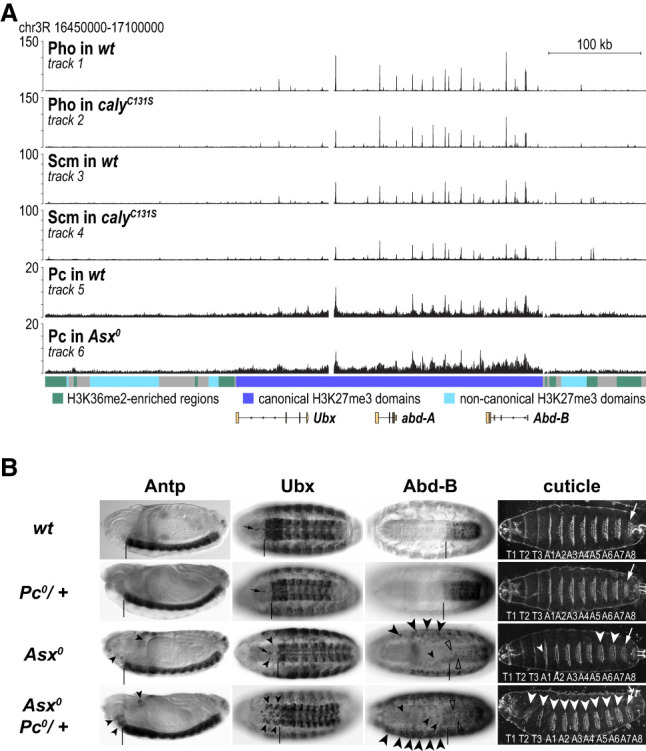
PRC1 binding to HOX gene chromatin is undiminished in PR-DUB mutants, but its repression capacity through chromatin compaction is impaired. (*A*) ChIP-seq profiles of Pho, Scm, and Pc in wild-type (wt) and PR-DUB mutant embryos at a chromosomal interval encompassing the *BX-C*. (*B*) Reduction of the levels of compaction-competent PRC1 exacerbates the Polycomb repression defects in *Asx*^*0*^ mutant embryos. Stage 16 embryos were stained for Antp, Ubx, or Abd-B, and embryonic cuticles; labeling of expression and morphological features are as in Figure 4A. In *Pc*^*0*^ heterozygous embryos (*Pc^0^/+*), expression of all three HOX proteins and cuticle morphology are indistinguishable from wild type (wt). *Asx*^*0*^ homozygous embryos that are heterozygous for *Pc*^*0*^ (*Asx^0^ Pc^0^/+*; fourth row) show more widespread misexpression of all three HOX genes (arrowheads) and more severe homeotic transformations of abdominal and thoracic segments (white arrowheads) compared with *Asx*^*0*^ homozygotes (third row).

We next analyzed the expression levels and genomic binding profiles of different PcG proteins in *Asx*^*0*^ or *caly*^*C131S*^ mutant embryos. The protein levels of diverse PhoRC, PRC1, and PRC2 subunits in *Asx*^*0*^ mutants were unaltered compared with wild type (Supplemental Fig. S11A). The genome-wide binding profiles of the PhoRC subunit Pho or of the PRC1 subunits Pc and Scm in PR-DUB mutants were also comparable with those in wild type (Fig. 6A; Supplemental Fig. S11B). In particular, the mutants showed no obvious reduction of PcG protein binding at HOX gene PREs, and also the low-level association of the Pc protein across canonical H3K27me3 domains appeared undiminished (Fig. 6A). This low-level Pc ChIP signal likely reflects direct interaction of Pc with H3K27me3-modified nucleosomes, which, as shown above (Fig. 3), are also present at undiminished levels across HOX gene chromatin in PR-DUB mutants. In conclusion, the increased H2Aub1 levels therefore do not interfere with the recruitment of PcG proteins to PREs, the ability of PRC2 to trimethylate H3K27me3 on nucleosomes in the flanking chromatin, or the ability of PRC1 to interact with these H3K27me3-marked nucleosomes. High H2Aub1 levels instead seem to interfere with a subsequent step of the Polycomb repression mechanism.

### Gene repression through PRC1-mediated chromatin compaction is compromised by high H2Aub1 levels

The biochemical and genetic evidence in *Drosophila* argues that PRC1 represses its target genes by compacting the nucleosome arrays that package these genes (Francis et al. 2001, 2004; King et al. 2005). Considering that H2Aub1 disrupts chromatin fiber folding in vitro (Fig. 5), an attractive mechanistic explanation for the defect in Polycomb repression in these mutants could be that H2Aub1 interferes with chromatin compaction by PRC1. To functionally test this hypothesis, we probed whether the defects in Polycomb repression in PR-DUB mutants would be further exacerbated in animals with impaired PRC1 chromatin compaction activity but intact E3 ligase activity. To this end, we used animals with a reduced dosage of Pc, the PRC1 subunit that is thought to promote chromatin compaction by enabling interactions of PRE-tethered PRC1 with H3K27me3-modified nucleosomes in the flanking chromatin. In embryos that are heterozygous for a *Pc*-null mutation (*Pc^0^/+*), HOX gene expression and the embryonic cuticle pattern are indistinguishable from wild-type embryos (Fig. 6B). However, in *Asx*^*0*^ mutant embryos, the same reduction in the dosage of the Pc protein (i.e., in *Asx^0^/Asx^0^; Pc^0^/+* embryos) resulted in markedly more extensive misexpression of Antp, Ubx, or Abd-B compared with *Asx*^*0*^ mutant embryos with wild-type levels of Pc (Fig. 6B). Consistent with this, *Asx^0^/Asx^0^; Pc^0^/+* mutant embryos also showed more severe homeotic transformations than *Asx*^*0*^*/Asx*^*0*^ single mutants (Fig. 6B). In conclusion, these experiments are compatible with a scenario in which the high H2Aub1 levels at HOX genes in PR-DUB mutants disrupt transcriptional repression by antagonizing the chromatin compaction activity of PRC1.

## Discussion

In this study, we investigated the molecular function of H2Aub1 in developing *Drosophila* with a focus on elucidating why and how H2Aub1 deubiquitinase activity of PR-DUB is needed for Polycomb repression.

The following main conclusions can be drawn from the work reported in this study. First, the genomic H2Aub1 profile shaped by the antagonistic actions of PRC1 and PR-DUB is unexpectedly dynamic during development. This balance permits PRC1 to generate H2Aub1 domains at Polycomb target genes in early embryos, but subsequently, it is H2Aub1 deubiquitination by PR-DUB that dominates and shapes a near-uniform, low-level H2Aub1 landscape in late stage embryos and larvae. Second, the early H2Aub1 domains are required for the rapid establishment of H3K27me3 domains; however, at HOX genes, this pathway does not appear to be critical because, with a delay, PRC2 generates H3K27me3 domains at these loci also in animals lacking H2Aub1. In contrast, the formation of noncanonical H3K27me3 domains strictly depends on H2Aub1. Third, in wild-type *Drosophila*, H2Aub1 is no longer enriched at Polycomb target genes during developmental stages when the Polycomb machinery acts to repress transcription of these genes. Together with the finding that Polycomb repression appears intact in animals lacking H2Aub1, this corroborates that chromatin compaction but not H2Aub1 is central to the PRC1 repression mechanism. Fourth, H2Aub1 inhibits chromatin fiber folding of nucleosome arrays in vitro. H2Aub1 therefore directly impacts the structural organization of chromatin. Fifth, in PR-DUB mutants, excessive H2Aub1 accumulation at HOX genes increases DNA accessibility and disrupts Polycomb repression at these loci. High H2Aub1 levels at these target genes are therefore detrimental to Polycomb repression. Sixth, removal of PRC1 E3 ligase activity restores Polycomb repression in PR-DUB mutants, whereas reduction of PRC1 chromatin compaction activity exacerbates the repression defects. This suggests that PR-DUB preserves Polycomb repression by antagonizing persistent H2Aub1 deposition by PRC1 in order to allow effective chromatin compaction by PRC1.

### H2Aub1 interferes with nucleosome stacking and chromatin fiber folding

Atomic structures of diverse nucleosome arrays revealed that stacking interactions between the solvent-exposed surfaces of the histone octamers in *n* and *n+2* nucleosomes are a common feature (Schalch et al. 2005; Song et al. 2014; Garcia-Saez et al. 2018; Dombrowski et al. 2022). In vivo, chromatin does not exist as long regularly folded fibers, but direct contacts between *n* and *n+2* nucleosomes are also widely observed using sequencing-based approaches (Hsieh et al. 2015; Risca et al. 2017). Moreover, tomographic studies suggest that nucleosome stacking is a prevalent feature of chromatin organization in vertebrate nuclei (Ou et al. 2017; Cai et al. 2018). Here, our FRET measurements show that these stacking interactions are critical for folding of unmodified nucleosome arrays in solution and that the presence of ubiquitin at H2AK119 sterically prevents nucleosome–nucleosome stacking. In conclusion, our results show that in addition to providing a binding site for PRC2.2 and variant PRC1 complexes, H2Aub1 can also directly impact the structural organization of chromatin.

### Excessive H2Aub1 accumulation in HOX gene chromatin accounts for disruption of Polycomb repression in PR-DUB mutants

To relate the observations from these in vitro studies to the effects of H2Aub1 on chromatin compaction in vivo, it is important to consider the density of H2Aub1-modified nucleosomes within a given stretch of chromatin in cells. In late stage wild-type embryos, only a few percent of total H2A carry the ubiquitin modification (Scheuermann et al. 2010). Considering that H2Aub1 is globally distributed across the entire genome in these embryos, one would expect that only a small fraction of nucleosomes across any given stretch of chromatin contains H2Aub1. During the stages when Polycomb repression acts, nucleosome stacking in Polycomb target gene chromatin is therefore unlikely to be grossly impaired. In contrast, the 20-fold to 30-fold increase in H2Aub1 nucleosome density in HOX gene chromatin in late stage PR-DUB mutant embryos could interfere with regular nucleosome stacking. The increased DNA accessibility at several Polycomb target genes in PR-DUB mutants is consistent with a less compact chromatin organization at these loci.

Our data argue that the high H2Aub1 accumulation in PR-DUB mutants does not interfere with Polycomb complex binding to PREs or H3K27me3 deposition in the flanking chromatin. The enhancement of the Polycomb repression defects in PR-DUB mutants with reduced PRC1 dosage (i.e., in *Pc/+*; *Asx*^*0*^ animals) is consistent with a scenario in which the excess of H2Aub1 directly impacts the ability of target gene-associated PRC1 to compact chromatin. This conclusion is in contrast to the recent suggestion that defective Polycomb repression in PR-DUB mutant mouse ESCs might be caused by reduced H3K27me3 deposition at Polycomb target genes (Conway et al. 2021; Fursova et al. 2021). Specifically, these studies proposed that the genome-wide low increase in H2Aub1 levels in PR-DUB mutants would sequester PRC2.2 and thereby titrate it away from Polycomb target genes. Our data provide no support for such a scenario in *Drosophila*.

The morphological defects in PR-DUB mutants suggest that Polycomb repression is primarily disrupted at HOX genes. Moreover, the repression defects at HOX genes are remarkably tissue- and stage-specific and not as widespread as in the case of mutants lacking PRC1 or PRC2. Even at HOX genes, the high H2Aub1 levels therefore still permit the Polycomb machinery to sustain repression in a fraction of cells. A possible explanation could be that the high H2Aub1 nucleosome density antagonizes chromatin compaction by PRC1 but does not fully abrogate it, and consequently, only some but not all tissue-specific enhancers in HOX genes are able to overcome this residual repression.

### Balancing H2A ubiquitination and deubiquitination during embryogenesis

What is the molecular basis for the formation of very different genomic H2Aub1 profiles in early and late embryos? Absolute quantification of proteins revealed that the copy number of Sce molecules per nucleus is comparable in early and late stage embryos (Bonnet et al. 2019). In contrast, the copy numbers of Calypso and Asx proteins in nuclei from early stage embryos are very low, and both proteins are fivefold to sevenfold more abundant in nuclei from late stage embryos (Bonnet et al. 2019). The lower abundance of PR-DUB relative to PRC1 in 0- to 6-h-old embryos could therefore provide a simple mechanistic explanation why PRC1 is able to generate H2Aub1 domains at Polycomb target genes early but not late in embryogenesis. Alternative scenarios to explain the different H2Aub1 landscapes in early and late embryos could be modulation of PRC1 or PR-DUB activity or differences in genomic targeting of these complexes during the different stages of embryogenesis.

### The role of H2Aub1 in H3K27me3 domain formation

The formation of the H3K27me3 landscape is thought to rely on the combined action of PRC2.1 and PRC2.2, whereby the relative contributions by these two complexes vary at different genomic locations (Laugesen et al. 2019; Yu et al. 2019). Here, we used H2Aub1-deficient embryos that lack the nucleosomal binding site specific for PRC2.2 (Kasinath et al. 2021). H2Aub1-deficient embryos show a genome-wide reduction of the H3K27me3 profile that is particularly pronounced in early embryos. During this stage of development, H2Aub1 and PRC2.2 therefore contribute significantly to H3K27me3 domain formation, analogously to what has been reported in mouse ESCs (Blackledge et al. 2020; Tamburri et al. 2020). Intriguingly, in later stage H2Aub1-deficient embryos, regular domains with near-normal coverage of H3K27me3 appear at HOX and many other canonical Polycomb target genes. This recovery of H3K27me3 domains explains why H2Aub1-deficient animals do not show the repression defects associated with loss of PRC2 activity. The recovery of near-normal levels of H3K27me3 at canonical H3K27me3 domains in late stage H2Aub1-deficient *Drosophila* embryos argues that the system, at least in flies, is considerably more plastic than anticipated.

### Repression by PRC1 without H2Aub1

A simple straightforward finding here was that H2Aub1 is not enriched at Polycomb target genes during the embryonic and larval stages when PRC1 is critically required to repress these genes. This is consistent with the finding that *Sce*^*I48A*^ mutants show no Polycomb mutant phenotypes and no general transcriptional deregulation of Polycomb target genes in late stage embryos. Together, these observations all argue against a critical role of H2Aub1 in the actual repression mechanism by which PRC1 blocks transcription. Identifying the reason why *Sce*^*I48A*^ or *H2A*^*K117R/K118R/K121R/K122R*^ mutants die as late stage embryos will require further studies, including experiments to investigate whether the low uniform levels of H2Aub1 across the genome might have an essential function in a process other than transcriptional regulation.

## Materials and methods

### Generation of the *caly*^*C131S*^ allele

The *caly*^*C131S*^ mutant allele was generated by scarless CRISPR–Cas9 technology (https://flycrispr.org/scarless-gene-editing). The genomic sequence of the engineered *caly*^*C131S*^ allele is shown in Supplemental Figure S2A.

### Generation of *caly*^*C131S*^ ^*m-z-*^ embryos

The conditionally excisable genomic >*caly*^+^> rescue transgene containing chr2R sequences 16,190,220–16,198,133 (dm6 reference genome) was cloned into pUMR (Supplemental Fig. S2C) and integrated into the attP landing site VK33 (BDSC 24871). One copy of the >*caly*^+^> transgene rescue*d caly*^*C131S*^ homozygotes into viable and fertile flies. Excision of the >*caly*^+^> transgene in the germline of *caly*^*C131S*^ heterozygous parents and identification of *caly*^*C131S*^ ^*m-z-*^ embryos are described in Supplemental Figure S2D.

### *Drosophila* strains and genotypes

See Supplemental Table S1 for detailed genotypes used in every experiment.

### Antibodies

Antibodies are listed in Supplemental Table S3.

### ChIP-seq analysis in *Drosophila* embryos and in larval tissues

A detailed protocol describing sample preparation, ChIP, library preparation, sequencing, and data processing is provided in the Supplemental Material.

### ATAC-seq and RNA-seq analysis

Detailed ATAC-seq and RNA-seq protocols are provided in the Supplemental Material.

### Immunohistochemistry and immunofluorescence stainings

Embryos of the appropriate genotypes were fixed and stained with Antp, Ubx, and Abd-B antibodies following standard protocols. Imaginal discs from third instar larvae were stained with Antp or Ubx and Cy3-labeled secondary antibodies following standard protocols. For clonal analysis, clones were induced by heat shock-induced expression of Flp recombinase in the genotypes listed in Supplemental Table S1.

### smFRET observation of chromatin conformation

H2Aub_SS_ was prepared according to Chatterjee et al. (2010), Fierz et al. (2011), and Debelouchina et al. (2017) and used to assemble histone octamers using purified human histones and, if required, recombinant H1.2 (for detailed protocols, see the Supplemental Material). Chromatin DNA containing fluorescent dyes was prepared and assembled into chromatin fibers as described previously (Kilic et al. 2018b). Flow chambers for TIRF experiments were assembled as described previously (Kilic et al. 2018b). Single-molecule measurements were performed on a micromirror TIRF system (MadCityLabs) (Kilic et al. 2018b). For each experiment, the flow chambers were incubated with 50 μL of 0.2 mg/mL neutravidin for 5 min, followed by immobilization of 0.5–2 μL of 5–20 ng/μL chromatin fibers. The buffer was exchanged to imaging buffer (40 mM KCl, 50 mM Tris-HCl, 2 mM Trolox, 2 mM nitrobenzyl alcohol [NBA], 2 mM cyclooctatetraene [COT], 10% glycerol, 3.2% glucose) supplemented with GODCAT (100× stock solution: 165 U/mL glucose oxidase, 2170 U/mL catalase). For imaging, ∼2000 frames were acquired with 532-nm laser excitation and 100-msec integration time. Acquired movies were analyzed using custom-written scripts in Matlab (MathWorks) as described before (Kilic et al. 2018b). From traces of donor (F_D_) and acceptor (F_A_) fluorescence emission intensity, FRET efficiency (E_FRET_) traces were calculated as follows:

$$E_{FRET}=\frac{F_{A}-\text{β}F_{D}}{F_{A}-\text{β}F_{D}+\gamma F_{D}}\;\mathrm{and}\;\gamma =\frac{\Delta F_{A,bleach}}{\Delta F_{D,bleach}},$$

with β = 0.161 and γ = 0.436. From fluorescence time traces, E_FRET_ histograms were constructed and fitted using two Gaussian functions:

$$y=\sum_{i=1-2}A_{i}e^{-\frac{1}{2}{\left(\frac{x-c_{i}}{d_{i}}\right)}^{2}}.$$

For all fit results, see Supplemental Table S2.

### Nuclear extracts for Western blot analysis

Total nuclear extracts were prepared by purifying nuclei from 21- to 24-h-old embryos that were quantified as described in Bonnet et al. (2019) and resuspended in appropriate volumes of SDS sample buffer. Extracts were sonicated in a Bioruptor (Diagenode) for eight cycles of 30 sec on/30 sec off at high-power mode and incubated for 5 min at 75°C, and insoluble material was removed by centrifugation.

### Data availability

Genomic data have been deposited in GEO under accession number GSE210236.

## Supplementary Material

Supplemental Material
