# Supplementary material for: PR-DUB preserves Polycomb repression by preventing excessive accumulation of H2Aub1, an antagonist of chromatin compaction
Source: Genes Dev. 2022 Oct 1;36(19-20):1046–61. doi: 10.1101/gad.350014.122 (PMC9744231; doi:10.1101/gad.350014.122)
Supplement: Supplemental Material [file supp_36_19-20_1046__DC1.html]

PR-DUB preserves Polycomb repression by preventing excessive accumulation of H2Aub1, an antagonist of chromatin compaction — PR-DUB preserves Polycomb repression by preventing excessive accumulation of H2Aub1, an antagonist of chromatin compaction — Supplemental Material 

# PR-DUB preserves Polycomb repression by preventing excessive accumulation of H2Aub1, an antagonist of chromatin compaction

## Supplemental Material

- Supplemental\_Material.pdf
