# Supplementary material for: PR-DUB preserves Polycomb repression by preventing excessive accumulation of H2Aub1, an antagonist of chromatin compaction
Source: Genes Dev. 2022 Oct 1;36(19-20):1046–61. doi: 10.1101/gad.350014.122 (PMC9744231; doi:10.1101/gad.350014.122)
Supplement: Supplemental Material [file supp_gad.350014.122_Supplemental_Material.pdf]

Supplemental Material

PR-DUB preserves Polycomb repression by preventing excessive accumulation of H2Aub1, an antagonist of chromatin compaction

Jacques Bonnet, Iulia Boichenko, Reinhard Kalb, Mathilde LeJeune, Svetlana Maltseva, Mattia Pieropan, Katja Finkl, Beat Fierz and Jürg Müller

Supplemental Material contains:

Supplemental experimental procedures

Supplemental Figures 1-11

Supplemental Table S1-S3

Supplemental References

Supplemental experimental procedures

ChIP-seq analysis in *Drosophila* embryos and in larval tissues

Embryo collection, chromatin preparation, and ChIP

0-6 hrs, 13-17 hrs and 21-24 hrs old wildtype, *Sce*^{I48A} or *caly*^{C131S} embryos as well as 21-24 hrs old *Asx*^{0} mutant embryos (see **Table S1** for details about the genotypes) were dechorionated, quick-frozen in liquid N2 and stored at -80˚C. Chromatins were prepared as described in Finogenova et al. (Finogenova *et al*, 2020) and 500 ng of chromatin were used for each ChIP experiment. 100 ng of an independently prepared batch of *D. pseudoobscura* chromatin were spiked-in in each ChIP experiment (prior to the addition of the antibody) for subsequent normalization of the ChIP-seq datasets. The ChIP protocol was then performed as described in Bonnet et al. (Bonnet *et al*, 2019). ChIP on hand-dissected wing and 3rd leg / haltere imaginal disc tissues from 3rd instar Orengo R larvae was performed as described in Laprell et al. (Laprell *et al*, 2017).

Library preparation and sequencing

Library preparation for sequencing was performed with Ovation® Ultralow System V2 (NuGEN, PART NO. 0344). Illumina systems (NextSeq 500) were used for paired-end DNA sequencing. BCL raw data were converted to FASTQ data and demultiplexed by bcl2fastq Conversion Software (Illumina). All reads were aligned using STAR (Dobin *et al*, 2013) to the *D. melanogaster* dm6 genome assembly (Dos Santos *et al*, 2015) and to the *D. pseudoobscura* dp3 genome assembly (Nov. 2004, FlyBase Release 1.03). Only sequences that mapped uniquely to the genome with a maximum of two mismatches were considered for further analyses.

Normalization of ChIP-seq datasets

The proportion of *D. pseudoobscura* reads as compared to *D. melanogaster* reads in input and in samples was used to normalize the ChIP-seq datasets of histone marks in embryos (See

Supplementary file 2 from Finogenova et al. (Finogenova *et al*, 2020) for more details). ChIP performed using antibodies against Polycomb group proteins or the RNA Pol II, and ChIP from larval tissues were normalized based on the total number of reads in each dataset.

Identification of H3K36me2- and H3K27me3-enriched regions

The Bioconductor STAN-package (Zacher *et al*, 2017) was used to define the location of H3K36me2- and H3K27me3-enriched regions. The seven chromosome arms (X, 2L, 2R, 3L, 3R, 4 and Y) defined in the dm6 genome assembly were segmented in 200 bp bins. STAN annotated each of these bins into 1 of 4 states based on the number of H3K27me3, H3K27me2, H3K36me2 ChIP-seq reads and the number of input reads overlapping with each bin in 21-24 hrs old wildtype embryos. These 4 genomic states corresponded to: 'H3K36me2-enriched' regions; 'H3K27me3-enriched' regions, 'other' regions and 'no input' regions (for simplification, these last two types of regions were merged under the name 'other'). The Poisson Lognormal distribution was selected and fitting of hidden Markov models was performed with a maximum number of 100 iterations. Two consecutive H3K27me3-enriched regions separated by less than 7000 bp were fused. Only stretches larger than 1000 bp were called H3K36me2-enriched regions. H3K27me3-enriched regions were subsequently divided into canonical and non-canonical H3K27me3 domains (canonical H3K27me3 domains correspond to the subset of H3K27me3-enriched regions with high H3K27me3 coverage, low H3K27me2 coverage and which overlap with one or more Pho peaks; see **Figure S1** for details).

Calculation of read coverage on gene bodies

ChIP-seq read coverages across gene bodies were computed on genomic intervals starting 750 bp upstream transcription start sites and ending 750 bp downstream transcription termination sites. Read coverage is defined as the normalized number of mapped reads per million reads from a ChIP-seq dataset divided by the number of mapped reads per million reads from the corresponding input dataset across a genomic region. Among the *D. melanogaster* FlyBase genes, 9530 are overlapping with H3K36me2-enriched regions, 640 are overlapping with

canonical H3K27me3 domains, 1795 are overlapping with non-canonical H3K27me3 domains and 5764 are localized in other genomic regions, in 21 to 24 hrs old embryos. Gene bodies overlapping with several types of chromatin domains were assigned to: non-canonical H3K27me3 domains in case of an overlap with such a domain; canonical H3K27me3 domains in case of an overlap with such domain and non with a non-canonical H3K27me3 domain; H3K36me2-enriched regions if there is an overlap with such a region and non with a H3K27me3-enriched region.

Identification of Pho, Scm and RNA PolII bound regions

Peak calling for Pho, Scm (from 13-17 hrs old wildtype embryos) and PolII S5P (from 21-24 hrs old wildtype embryos) ChIP-seq datasets was performed using MACS 2.2.6.

ATAC-seq and RNA-seq analysis

ATAC-seq

Batches of 120 13-17 hrs old wildtype or *caly*^{C131S} embryos were collected, dechorionated and homogenized in ice-cold Nu1 buffer (15 mM HEPES pH 7.6, 20 mM KCl, 5 mM MgCl2, 0.5 mM EDTA pH 7.9, 0.5 mM EGTA pH 7.9, 20% glycerol, 350 mM sucrose and 0.1% NP40, complemented with protease inhibitors) and the homogenates were filtered through Miracloth tissue (Calbiochem). Nuclei were washed in ATAC Lysis Buffer from the ATAC-Seq kit (Active Motif (#53150)) and the washed pellets were snap frozen in liquid nitrogen and stored at -80˚C. Before the last centrifugation step, 5% of the purified nuclei were collected and diluted into a final volume of 100 µL of PBS for genomic DNA extraction to determine the number of purified nuclei in each sample. Proteins were digested by addition of 5 µg of Proteinase K for 1 hrs at 50˚C and the enzyme was then heat inactivated at 94 ˚C for 10 minutes. qPCR analysis was then performed to determine the copy number of four different genomic DNA regions (coordinates in dm6: chr2R: 5 843 708 – 5 843 838, chr2R: 5 640 224 – 5 640 348, chr2R: 19 447 639 – 19 447 733 and chr3L: 4 140 113 – 4 140 244) in these

samples, using defined copy numbers of synthesized linear DNA fragments (Twist Bioscience) containing these four genomic DNA regions, as quantification standard in parallel qPCR reactions. Frozen nuclear pellets were then resuspended in ATAC Lysis Buffer to get a concentration of 25000 nuclei per μL and 1 μL of the suspension was used for the tagmentation reaction. Tagmentation reaction, DNA purification and PCR Amplification of Tagmented DNA was performed with the ATAC-seq kit (Active Motif (#53150)), following the instructions of the manufacturer. Sequencing and read mapping was performed as described for the ChIP-seq analysis. Different ATAC-seq samples were normalized based on the total number of reads in each dataset. ATAC-seq signal is defined as the normalized number of mapped reads per million reads from an ATAC-seq dataset across a genomic region.

Definition of regions with low, intermediate and high DNA accessibility

The Bioconductor STAN-package ((Zacher *et al*, 2017), see above) was used to annotate the genome into 4 states corresponding to regions with low, intermediate and high DNA accessibility, and 'no input' regions (these non-unique DNA sequences are represented in white on the bar track in **Figure 5H** and **Figure S10A**). The genome was segmented in 200 bp bins and STAN annotated each of them into 1 of the 4 states based on the number of overlapping reads from 13-17 hrs old wildtype embryo ATAC-seq datasets and from a DNA input dataset (as a control to identify genomic regions with non-unique DNA sequences). The Poisson Lognormal distribution was selected and fitting of hidden Markov models was performed with a maximum number of 100 iterations. The overlap between these DNA accessibility-based regions and the four types of chromatin domains (see above and **Figure S1**), was then determined. For regions defined based on DNA accessibility that are overlapping with several types of chromatin domains, the same logic described for gene bodies was also applied (see above). Among the regions with low or intermediate DNA accessibility, only those larger than 1kb were considered for further analyses. 4118, 2315, 863, 26, 38, 59, and 9790 regions with low DNA accessibility are respectively located in H3K36me2-enriched domains, non-canonical

H3K27me3 domains, canonical H3K27me3 domains, *Abd-B* regulatory regions, *Ubx* and *abd-A* regulatory regions, *Antennapedia* complex and other genomic regions. 5242, 1824, 519, 8, 28, 73, and 7365 regions with intermediate DNA accessibility are respectively located in H3K36me2-enriched domains, non-canonical H3K27me3 domains, canonical H3K27me3 domains, *Abd-B* regulatory regions, *Ubx* and *abd-A* regulatory regions, *Antennapedia* complex and other genomic regions. 5508, 1279, 529, 20, 28, 53, and 5393 regions with high DNA accessibility are respectively located in H3K36me2-enriched domains, non-canonical H3K27me3 domains, canonical H3K27me3 domains, *Abd-B* regulatory regions, *Ubx* and *abd-A* regulatory regions, *Antennapedia* complex and other genomic regions.

RNA-seq from single embryos

15-16 hrs old (stage 16) wildtype, Asx^{0} or Sce^{I48A} embryos were dechorionated and transferred on a glass slide in drops of Halocarbon oil 700. Their morphology was analyzed and pictured by differential interference contrast microscopy. Embryos were then disrupted in 40 µL of Trizol (ThermoFisher Scientific), complemented with 8 µg of UltraPure glycogen (ThermoFisher Scientific). 960 µL of Trizol were then added to each sample and RNA purification was performed following the instructions from the manufacturer. Each RNA pellet was resuspended in 22 µL of DEPC-treated water (Roth). mRNA sequencing libraries were prepared with 10ng of total RNA of each sample using the NEBNext® Ultra™ II RNA Library Prep Kit for Illumina (NEB, E7770) with NEBNext® Poly(A) mRNA Magnetic Isolation Module (NEB, E7490), according to standard manufacturer's protocol. Total RNA and the final library quality controls were performed using Qubit™ Flex Fluorometer (Invitrogen, Q33327) and 2100 Bioanalyzer Instrument (Agilent, G2939BA) before and after library preparation. Paired-end sequencing was performed on Illumina NextSeq 500 with High Output Kit v2.5 (42bp - 42bp). BCL raw data were converted to FASTQ data and demultiplexed by bcl2fastq Conversion Software (Illumina). 12 wildtype, 8 Asx^{0} and 8 Sce^{I48A}

RNA-seq datasets were generated. *Drosophila* transcript (from release 6.35) quantification was performed from fastq files using Salmon (Patro *et al*, 2017). Abundances were summarized from transcript- to gene-level with the Bioconductor package tximeta (Love *et al*, 2020) and differential expression analysis was performed using the DESeq2 package (Love *et al*, 2014). A first differential expression analysis with all datasets from a mutant genotype and all wildtype datasets was performed to identify by principle component analysis, a subset of comparable datasets originating from wildtype and mutant embryos of equivalent developmental stage. This selection was further validated by looking at the embryonic morphology. This led to the selection of 5 Asx^{0} with 3 wildtype embryos and 4 Sce^{I48A} with 4 wildtype embryos to perform the final differential expression analysis. Among the about 11500 analyzed genes, about 7800, 350, 1000 and 2300 are respectively located in H3K36me2-enriched regions, canonical and non-canonical H3K27me3 regions, and in other genomic regions.

Material preparation for smFRET observation of chromatin conformation

Synthesis of $H2Aub_{SS}$

$H2Aub_{SS}$ was prepared following (Chatterjee *et al*, 2010; Fierz *et al*, 2011; Debelouchina *et al*, 2017). In short, ubiquitin (1-76) was cloned in frame with a single-chain version of the split-intein Npu, containing a C-terminal mutation of the catalytic asparagine and the +1 cysteine (in the extein) to alanine:

Ub-Npu(AA):

MQIFVKTLTGKTITLEVEPSDTIENVKAKIQDKEGIPPDQQRLIFAGKQLEDGRTLSDY

NIQKESTLHLVLRLRGG-

CLSYETEILTVEYGLLPIGKIVEKRIECTVYSVDNNGNIYTQPVAQWHDRGEQEVFEY

CLEDGSLIRATKDHKFMTVDGQMLPIDEIFERELDLMRVDNLPNIKIATRKYLGKQN

VYDIGVERDHNFALKNGFIASAAFNHHHHHH

The construct was expressed in BL21(DE3)plysS cells (induction for 4h with 0.5 mM IPTG),

the cells were lysed and the Ub-intein fusion was purified over a Ni:NTA affinity column. The protein was eluted with 600 mM imidazole in 20 mM Tris-HCl, pH 6.8 and 200 mM NaCl elution buffer. 25 mM cysteamine and 50 mM TCEP were added and the intein cleavage was let to proceed overnight (**Figure S6A**). Ubiquitin-SH was purified by preparative RP-HPLC using a gradient of 0-70% B, analyzed by analytical RP-HPLC (**Figure S6B**) and electrospray liquid chromatograpy mass spectroscopy (ESI-LCMS) (**Figure S6C**).

Conversely, histone H2A containing a K119C mutation was expressed in BL21(DE3)plysS cells (6L, induced for 4h with 0.5 mM IPTG), the cells were lysed and inclusion bodies were purified using two washes with wash buffer (20 mM Tris-HCl, pH 7.5, 200 mM NaCl, 1 mM EDTA, 1 mM PMSF) supplemented with 1 % Triton X-100, and two washes with neat wash buffer. The histone was then resolubilized in 20 mL of unfolding buffer (7 M Guanidium hydrochloride, 20 mM Tris-HCl, 10 mM DTT, pH 7.5), dialyzed into buffer A (6 M urea, 10 mM Tris-HCl, 1mM EDTA, 100 mM NaCl, 1 mM DTT, 0.2 mM PMSF, pH 7.5), followed by purification by cation exchange (using a HiTrap SP HP 5 mL column) eluted with a gradient of 10-100% B (6 M urea, 10 mM Tris-HCl, 1mM EDTA, 1 M NaCl, 1 mM DTT, 0.1 mM PMSF, pH 7.5). Collected fractions were further purified using C18 preparative RP-HPLC. Pure fractions were combined, lyophilized and stored at -20 °C.

To generate H2AK119-TNP (an asymmetric disulfide with 2-thio-5-nitropyridine), a solution of 2,2 -dithiobis(5-nitropyridine) DTNP (20.7 mg, 66.6 µmol, 30 eq) in 2.52 mL of 3:1 (v/v) CH3COOH:MQ H2O was added to H2A K119C (30.0 mg, 2.22 µmol, 1.0 eq). The reaction mixture was incubated at 25 °C for 18 h, RP-HPLC was used to observe the reaction progress (**Figure S6D**) and the product was purified by C18 preparative RP-HPLC eluting with a gradient of 40-68% B over 50 min to give 9 (18.8 mg, 1.33 µmol, 62%), resulting in pure H2AK119-TNP (**Figure S6E-F**).

For the synthesis of H2Aub$_{SS}$, a solution of H2AK119-TNP (1.85 mg, 0.130 µmol, 1.0 eq) in 771 µL of reaction buffer (1 M HEPES, 6 M Gn-HCl, pH 6.91) was added to ubiquitin-

SH (1.70 mg, 0.200 μmol, 1.5 eq) (**Figure S6G**). The reaction was incubated at 25 °C for 30 min with continuous shaking. The reaction mixture was purified by semi-preparative RP-HPLC eluting with a gradient of 45-65% B over 40 min to give H2Aub$_{SS}$ (2.4 mg, 0.106 μmol, 83%), which was analyzed by RP-HPLC and ESI-LCMS (**Figure S6H-I**).

Histone octamer refolding

In a typical octamer refolding reaction, 0.5-1.5 mg of pure lyophilized human histones were taken up in unfolding buffer (6 M Gn-HCl, 20 mM Tris-HCl, pH 7.5). The exact concentration was determined by UV spectroscopy, using the following extinction coefficients: ε_{280} nm, H2Aub$_{SS}$ = 5960 $M^{-1}cm^{-1}$, ε_{280} nm, H2B = 7450 $M^{-1}cm^{-1}$, ε_{280} nm, H3 = 4470 $M^{-1}cm^{-1}$, ε_{280} nm, H4 = 5960 $M^{-1}cm^{-1}$. Histones were combined in equimolar ratios, at a concentration of ~ 1 mg/mL. Octamers were refolded by dialysis against refolding buffer (2 M NaCl, 10 mM Tris-HCl, 1 mM EDTA, pH 7.5). The refolded octamers were subsequently purified by size exclusion S200 10/300 chromatography column eluting with refolding buffer (**Figure S6J**). Collected fractions were analyzed by SDS-PAGE, and octamer containing fractions were pooled and concentrated. Glycerol was added to a final concentration of 50% (v/v) and octamers were stored at -20 °C.

Production of labelled chromatin DNA

Chromatin DNA was produced as shown in **Figure S7A** and described in (Kilic *et al*, 2018). Briefly, recombinant pieces recP1 and recP5, flanked by DraIII and BsaI restriction enzyme sites were expressed in bacterial cells and purified via PEG-precipitation at the indicated PEG percentages (see **Figure S7B-D** for 30 bp linker DNA, and **Figure S7E-G** for 50 bp linker DNA). The sequence for the repeating unit for both 30 and 50 bp linker DNA sequences are given below (601 sequence in grey bold (Thåström *et al*, 1999)), donor labelling position in red, acceptor labelling position in violet).

30 bp linker:

CTGGAGAATCCCGGTGCCGAGGCCGCTCAATTGGTCGTAGACAGCTCTAGC ACCGCTTAAACGCACGTACGCGCTGTCCCCCGCGTTTTAACCGCCAAGGGG ATTACTCCCTAGTCTCCAGGCACGTGTCAGATATATACAAGATCCCTAGATCC ATGGAGTACTTACGCGGCCGCC

50 bp linker:

CTGGAGAATCCCGGTGCCGAGGCCGCTCAATTGGTCGTAGACAGCTCTAGC ACCGCTTAAACGCACGTACGCGCTGTCCCCCGCGTTTTAACCGCCAAGGGG ATTACTCCCTAGTCTCCAGGCACGTGTCAGATATATACAAGATCCGCATGTAT TGAACAGCATGATCAGTACTATGGACCCTATACGCGGCCGCC

Fragments P2, P3 and P4 were produced by PCR reactions using fluorescently labeled primers using indicated dyes (**Figure S7A**), for P2 and P4, and purified. Each piece was digested with the restriction enzymes BsaI-HF and DraIII-HF, resulting in non-palindromic unique overhangs. Digested DNA fragments were purified by PEG precipitation. For preparative ligations, 30-60 pmol of each DNA piece was used to generate the intermediates in combined volumes of 200-400 μL: P2 was ligated to P1 in 20% excess for 2 h in 1x T4 DNA ligase buffer with 60 U of ligase, then P3 was added in 20% excess relative to P2 and ligation allowed to proceed overnight. At the same time, P4 was ligated to P5 in 20% excess for 12-16 h (**Figure S7H,I,** I. DNA ligation). The pieces were purified by PEG precipitation using a stepwise (0.5% steps) increase in PEG from 7.0% to 8.0%. Pellets containing the purified desired chromatin DNA intermediates were redissolved in 60 μL TE buffer (10 mM Tris-HCl, 0.1 mM EDTA, pH 8.0), were pooled and stored for later ligations (**Figure S7H,I,** II. PEG precipitation). 15-35 pmol of the 6x601 intermediates were mixed using 5-10% excess P4-P5, the biotinylated

anchor was added as well as 1x T4 DNA ligase buffer with 60 U of ligase. The mixture was then left to ligate for 10-16 h (**Figure S7H,I**, III. DNA ligation). The formation of the product was confirmed by agarose gel electrophoresis and purified by step-wise PEG precipitation in the range 5.0-6.0% (**Figure S7H,I**, IV. PEG precipitation). The pellets were redissolved in TE(10/0.1) and analyzed by gel electrophoresis to pool the purified double-labeled array DNA.

Chromatin assembly

Chromatin assembly was performed as previously described (Kilic *et al*, 2018). Chromatin arrays were reconstituted on a scale of ~ 20 pmol (calculated based on nucleosome positioning sequences (NPS)). Fluorescently labelled chromatin array DNA containing FRET dyes and 30 or 50 bp linker DNA was combined with equimolar amounts of MMTV buffer DNA, NaCl was added to a final concentration of 2 M, followed by the addition of equimolar equivalents of histone octamers (either unmodified or containing H2Aub$_{SS}$). If indicated, H1.2 was added at this stage (equivalents are experimentally determined, usually 1-2 equivalents per nucleosome, the variation being due to uncertainties in concentration determination). The mixture was dialyzed with a gradient from TEK2000 buffer (10 mM Tris-HCl, 0.1 mM EDTA, pH 7.5, 2000 mM KCl) to TEK10 buffer (10 mM Tris-HCl, 0.1 mM EDTA, pH 7.5, 10 mM KCl) over 16 h. The dialyzed mixture was taken up in 200 mL TEK10 and further dialyzed for 1 h. The chromatin assembly mixtures were then centrifuged at 25,000 x g for 10 min and the supernatant was collected, and analyzed via native agarose gel-electrophoresis (**Figure S8A, F, G**). The concentration of the crude assembly was determined by UV-vis spectrometry. The quality of the chromatin assemblies were further assessed by ScaI-digestion of the arrays, liberating individual nucleosomes. Chromatin arrays were combined with an equal volume of 1 x CutSmart buffer (New England Biolabs) and 10 U of ScaI-HF restriction enzyme, followed by digestion for 4 h at 37 °C. ScaI digests were analyzed by native PAGE (**Figure S8B, F, H**). Only chromatin arrays that showed full nucleosomal occupancy in ScaI digest samples were

used for further experiments.

Purification of H1.2

H1.2 was expressed in BL21(DE3) cells after induction by 1mM IPTG for 3 h, followed by cell lysis in Lysis buffer (50 mM Tris-HCl, pH 8.0, 200 mM NaCl, 1 mM EDTA, 1 mM PMSF, 1 mM BME, 1x protease inhibitor cocktail). Cell lysates were cleared by centrifugation at 15000 x g for 10 min at 4 °C. H1.2 was purified by IEX (5 mL HiTrap SP HP, Cytiva) using a gradient 0-100% B (A: 20 mM Tris-HCl pH 8.0, 200 mM NaCl, 1 mM EDTA, 0.2 mM PMSF, B: 20 mM Tris-HCl pH 8.0, 1 M NaCl, 1mM EDTA, 0.2 mM PMSF) in 30 CV. Fractions were analyzed by SDS-PAGE (**Figure S8C**). Fractions containing H1.2 were combined, the buffer was exchanged to Size exclusion buffer (PD10, 20 mM Tris-HCl pH 8.0, 150 mM NaCl, 1 mM PMSF), concentrated, and subjected to size exclusion chromatography purification (S75 10/300, Cytiva) (**Figure S8D**). Pure fractions were combined, concentrated, quantified via UV-Vis, characterized by ESI-LCMS (**Figure S8E**), and stored in 20% glycerol at -80°C.

H1.2 sequence:

SETAPAAPAAAPPAEKAPVKKKAAKKAGGTPRKASGPPVSELITKAVAASKERSGVS
LAALKKALAAAGYDVEKNNSRIKLGLKSLVSKGTLVQTKGTGASGSFKLNKKAASG
EAKPKVKKAGGTKPKKPVGAAKKPKKAAGGATPKKSAKKTPKKAKKPAAATVTKK
VAKSPKKAKVAKPKKAAKSAAKAVKPKAAKPKVVKPKKAAPKKK

Flow chamber preparation

Flow chambers for TIRF experiments were prepared as previously described in the literature (Kilic *et al*, 2018). Briefly, borosilicate glass slides with 2 rows of 4 holes and borosilicate coverslips were cleaned by sonication for 20 min in ultra-pure H_2O, followed by acetone, ethanol and piranha solution (25% v/v H_2O_2 and 75% v/v H_2SO_4). Then, they were washed

with H_2O until reaching neutral pH, again sonicated in acetone for 10 min and then immersed in 3% v/v (3-aminopropyl)triethoxysilane in acetone for 20 min. Finally, slides and coverslips were washed in ultra-pure H_2O and dried with N_2. On each slide, four flow-chambers were assembled using strips of double-sided 0.12 mm tape and a coverslip. The chambers were sealed with epoxy glue and stored under vacuum at -20 °C until use.

Before measurements, the flow chambers were fitted with pipette tips in each of the 2 x 4 holes. Subsequently, 350 µL of 0.1 M tetraborate buffer at pH 8.5 was used to dissolve ~1 mg of biotin-mPEG (5000kDa) -SVA, and 175 µL from this was transferred to 20 mg mPEG (5000kDa) -SVA to generate a transparent clouding-point solution after 10 s of centrifugation. This was mixed to homogeneity and centrifuged for 10 s before 40-45 µL were loaded into each of the four channels in the flow chamber, incubated at RT for 2 h, after which the solution was washed out with degassed ultra-pure H_2O.

Figure S1

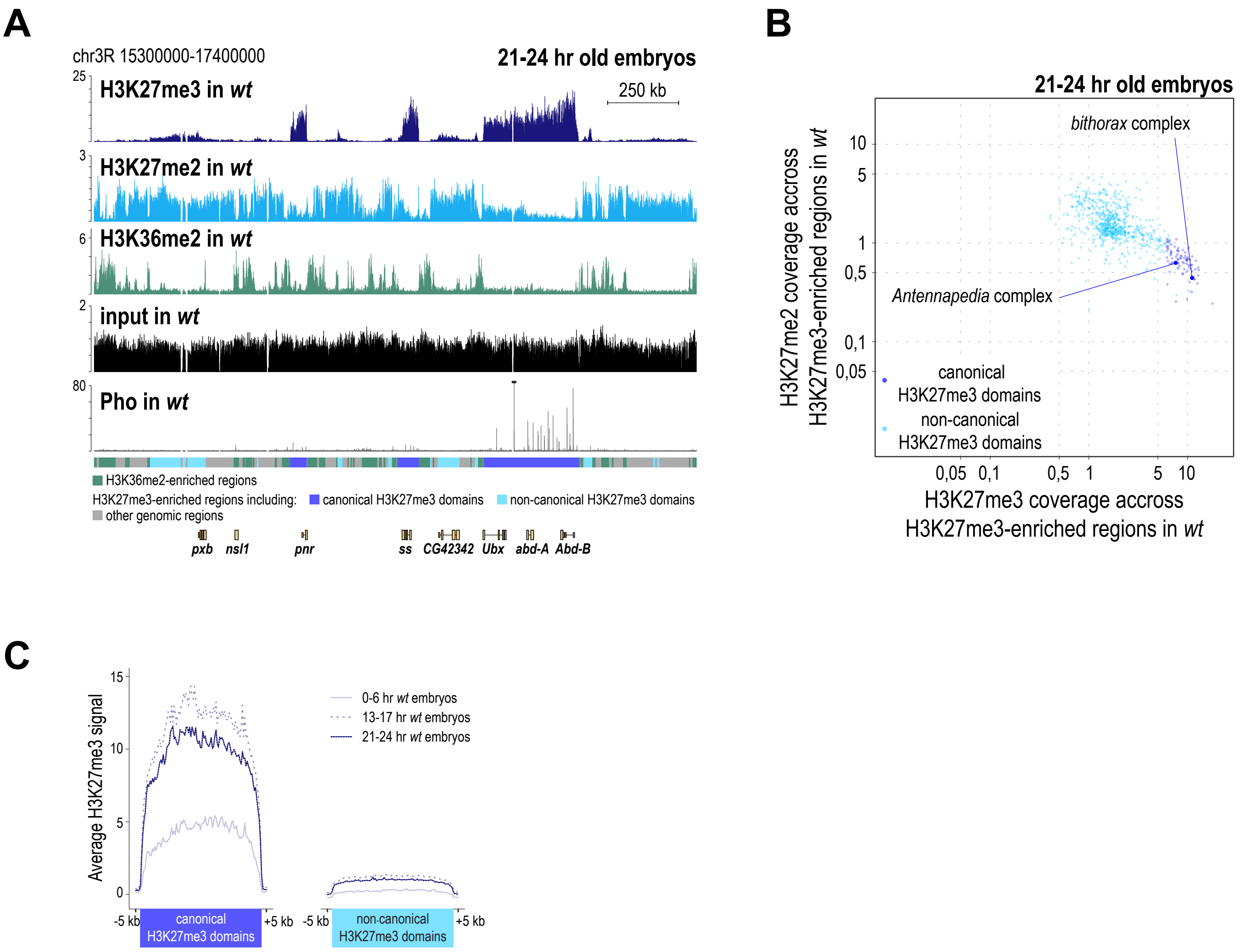

A
chr3R 15300000-17400000
21-24 hr old embryos
250 kb
H3K27me3 in wt
H3K27me2 in wt
H3K36me2 in wt
input in wt
Pho in wt
H3K36me2-enriched regions
H3K27me3-enriched regions including: canonical H3K27me3 domains non-canonical H3K27me3 domains
other genomic regions
pxb nsl1 pnr ss CG42342 Ubx abd-A Abd-B
B
21-24 hr old embryos
bithorax complex
Antennapedia complex
canonical H3K27me3 domains
non-canonical H3K27me3 domains
H3K27me2 coverage accross H3K27me3-enriched regions in wt
H3K27me3 coverage accross H3K27me3-enriched regions in wt
C
Average H3K27me3 signal
0-6 hr wt embryos
13-17 hr wt embryos
21-24 hr wt embryos
-5 kb
+5 kb
canonical H3K27me3 domains
non-canonical H3K27me3 domains

Figure S1

Definition of canonical and non-canonical H3K27me3 domains and of H3K36me2 domains by genome segmentation

(**A**) H3K27me3, H3K27me2, H3K36me2 and Pho ChIP-seq profiles and input profile in 21-24 hrs old wildtype (*wt*) embryos. Of these, the H3K27me3, H3K27me2, H3K36me2 and input datasets were used to perform genome segmentation and this defined 2507 H3K36me2-enriched regions spanning 29.8 Mb of the *1C* genome (green), 735 H3K27me3 enriched-regions spanning 18.7 Mb of the *1C* genome that, as described in (**B**), were then subdivided into canonical (dark blue) and non-canonical (light blue) H3K27me3 domains, and 3017 genomic regions without H3K36me2 or H3K27me3 enrichment that covered 89.1 Mb of the *1C* genome (grey). The genomic interval shown here corresponds to the interval used in the main figures 1-3 and 7.

(**B**) Subdivision of H3K27me3 enriched regions into canonical and non-canonical H3K27me3 domains. Scatter plots showing H3K27me3 and H3K27me2 read coverage across H3K27me3 enriched regions. Regions with high H3K27me3 coverage (> 6), low H3K27me2 coverage (< 1.1) and overlap with the center of at least 1 Pho peak were defined as canonical H3K27me3 domains (84 regions, covering 5.8 Mb of the *1C* genome, dark blue). These regions comprise the known Polycomb target genes, including the HOX genes in the *bithorax* and *Antennapedia* complexes. The remaining H3K27me3-enriched regions were defined as non-canonical H3K27me3 domains (651 regions, covering 12.9 Mb of the *1C* genome, light blue).

(**C**) Average H3K27me3 ChIP-seq profiles at canonical and non-canonical H3K27me3 domains in early, mid- and late-stage wildtype embryos.

Figure S2

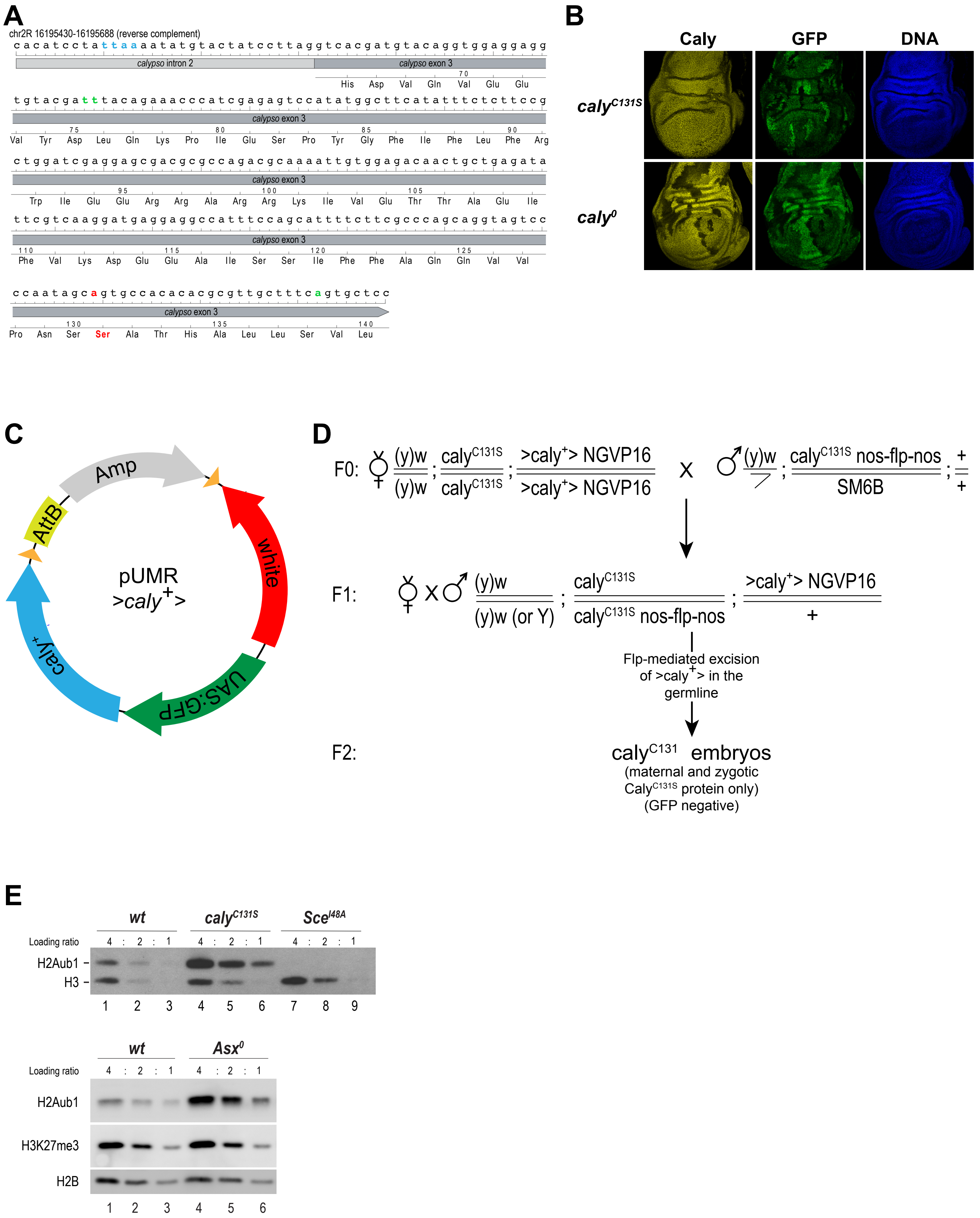
A
chr2R 16195430-16195688 (reverse complement)
cacatcctattaaaatatgtactatccttaggtcacgatgtacaggtggaggagg
calypso intron 2
calypso exon 3
His Asp Val Gln Val Glu Glu
tgtacgatttacagaaacccatcgagagtccatatggcttcatatttctcttccg
Val Tyr Asp Leu Gln Lys Pro Ile Glu Ser Pro Tyr Gly Phe Ile Phe Leu Phe Arg
ctggatcgaggagcgacgcgccagacgcaaaattgtggagacaactgctgagata
Trp Ile Glu Glu Arg Arg Ala Arg Arg Lys Ile Val Glu Thr Thr Ala Glu Ile
ttcgtcaaggatgaggaggccatttccagcattttcttcgcccagcaggtagtcc
Phe Val Lys Asp Glu Glu Ala Ile Ser Ser Ile Phe Phe Ala Gln Gln Val Val
ccaatagcagtgccacacacgcgttgctttcagtgctcc
Pro Asn Ser Ser Ala Thr His Ala Leu Leu Ser Val Leu
B
Caly
GFP
DNA
caly C131S
caly 0
C
Amp
AttB
white
pUMR
>caly+>
caly+
UAS:GFP
D
F0: (y)w / (y)w ; caly C131S / caly C131S ; >caly+> NGVP16 / >caly+> NGVP16 X (y)w / ; caly C131S nos-flp-nos / SM6B ; + / +
F1: X (y)w / (y)w (or Y) ; caly C131S / caly C131S nos-flp-nos ; >caly+> NGVP16 / +
Flp-mediated excision of >caly+> in the germline
F2: caly C131 embryos
(maternal and zygotic Caly C131S protein only)
(GFP negative)
E
wt
caly C131S
Sce I48A
Loading ratio
4 : 2 : 1
H2Aub1
H3
1 2 3 4 5 6 7 8 9
wt
Asx 0
H2Aub1
H3K27me3
H2B
1 2 3 4 5 6

Figure S2

Strategy used to generate *caly*^{C131S} catalytic-dead mutant embryos.

(**A**) Genomic sequence (chr2R 16195430-16195688, reverse complement) of the *caly*^{C131S} allele, including all edited nucleotides. In the process of CRISPR-Cas9 mediated genome editing (https://flycrispr.org/scarless-gene-editing/), homology-directed repair led to the integration of a *DsRed* marker cassette flanked by homology arms containing the desired DNA mutations. The substituted nucleotide mutating catalytic Cys1,31 in *caly* into Ser is labelled in red. Silent mutations abrogating the PAM sites of the two used gRNA for cleavage by Cas9 are labelled in green. Integration of the *DsRed* marker cassette, adjacent to the endogenous TTAA site (in blue) at the end of the second *caly* intron, enabled restoration of the genomic sequence after excision of the cassette with the PBac transposase.

(**B**) Wing imaginal discs from larvae with clones of *caly*^{C131S} homozygous mutant cells (top) or *caly*^{0} homozygous mutant cells (bottom) stained with antibodies against Calypso protein (yellow) and Hoechst (DNA) to label all nuclei. In both genotypes, clones were induced 72 hrs before analysis and mutant clone cells are marked by the absence of GFP. Note the loss of Calypso protein signal in the *caly*^{0} homozygous mutant cells and that the levels of Calypso^{C131S} protein in *caly*^{C131S} homozygous mutant cells are undiminished compared to wildtype cells.

(**C**) Map of the pUMR >*caly*$^{+}$> vector. AttB: *AttB-AttP* recombination site; Amp: *Ampicillin* resistance gene; white: *white* marker gene; UAS:GFP: *14xUAS*$_{Gal4}$*-nuclear GFP* marker cassette (Gambetta & Müller, 2014), *caly*$^{+}$: genomic *calypso* gene fragment (see Supplemental Experimental Procedures). The yellow arrowheads represent the FRT sites; FLP-mediated excision results in deletion of the cassette harboring *caly*$^{+}$ and the *GFP* and *white* marker genes.

(**D**) Crosses performed to generate *caly*$^{C131S\ m-\ z-}$ embryos. NGVP16: *nanos-Gal4-VP16*; nos-flp-nos: FLP recombinase coding sequence under the control of *nanos* regulatory sequences (Kaushal *et al*, 2021).

(**E**) Western blot analysis on serial dilutions of total nuclear extracts from 21-24 hrs old embryos shows that H2Aub1 bulk levels are about 4-fold higher in *caly*^{C131S} (top) or in *Asx*^{0} mutant embryos (bottom) compared to wild-type (*wt*). Loss of H2Aub1 signal in extracts from *Sce*^{I48A} mutant embryos (top) is shown as control. H3K27me3 bulk levels were unchanged in *Asx*^{0} mutant embryos.

Figure S3

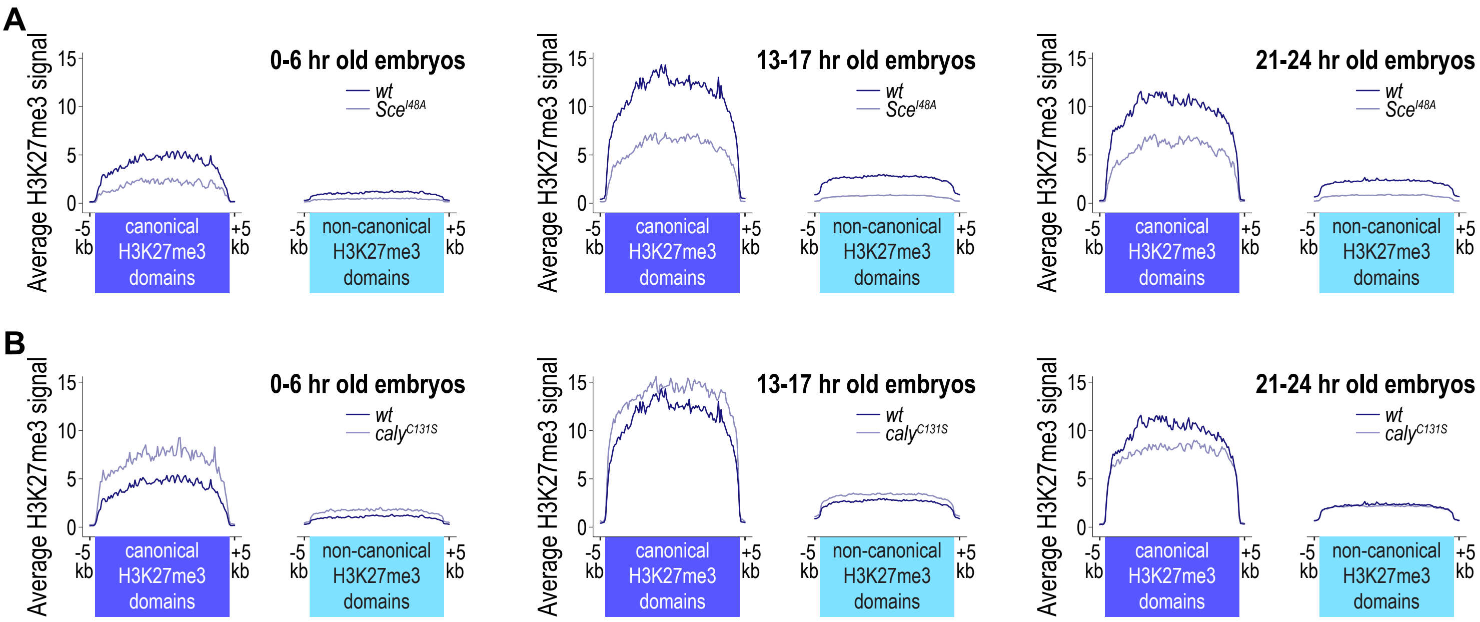
A
Average H3K27me3 signal
15
10
5
0
0-6 hr old embryos
13-17 hr old embryos
21-24 hr old embryos
wt
Sce^I48A
-5 kb
+5 kb
canonical H3K27me3 domains
non-canonical H3K27me3 domains
B
wt
caly^C131S

Figure S3

Meta-analyses of H3K27me3 in *Sce*^{I48A} and *caly*^{C131S} mutant embryos.

(**A**) Loss of H2Aub1 delays generation of canonical H3K27me3 domains and abolishes generation of non-canonical H3K27me3 domains. Average distribution profiles of H3K27me3 at canonical and non-canonical H3K27me3 domains in 0-6 hr, 13-17 hr and 21-24 hr old wildtype and *Sce*^{I48A} mutant embryos.

(**B**) Excess of H2Aub1 does not strongly affect the generation of any type of H3K27me3 domain. Average distribution profiles of H3K27me3 as in (**A**) but comparing wildtype and *caly*^{C131S} mutant embryos.

Figure S4

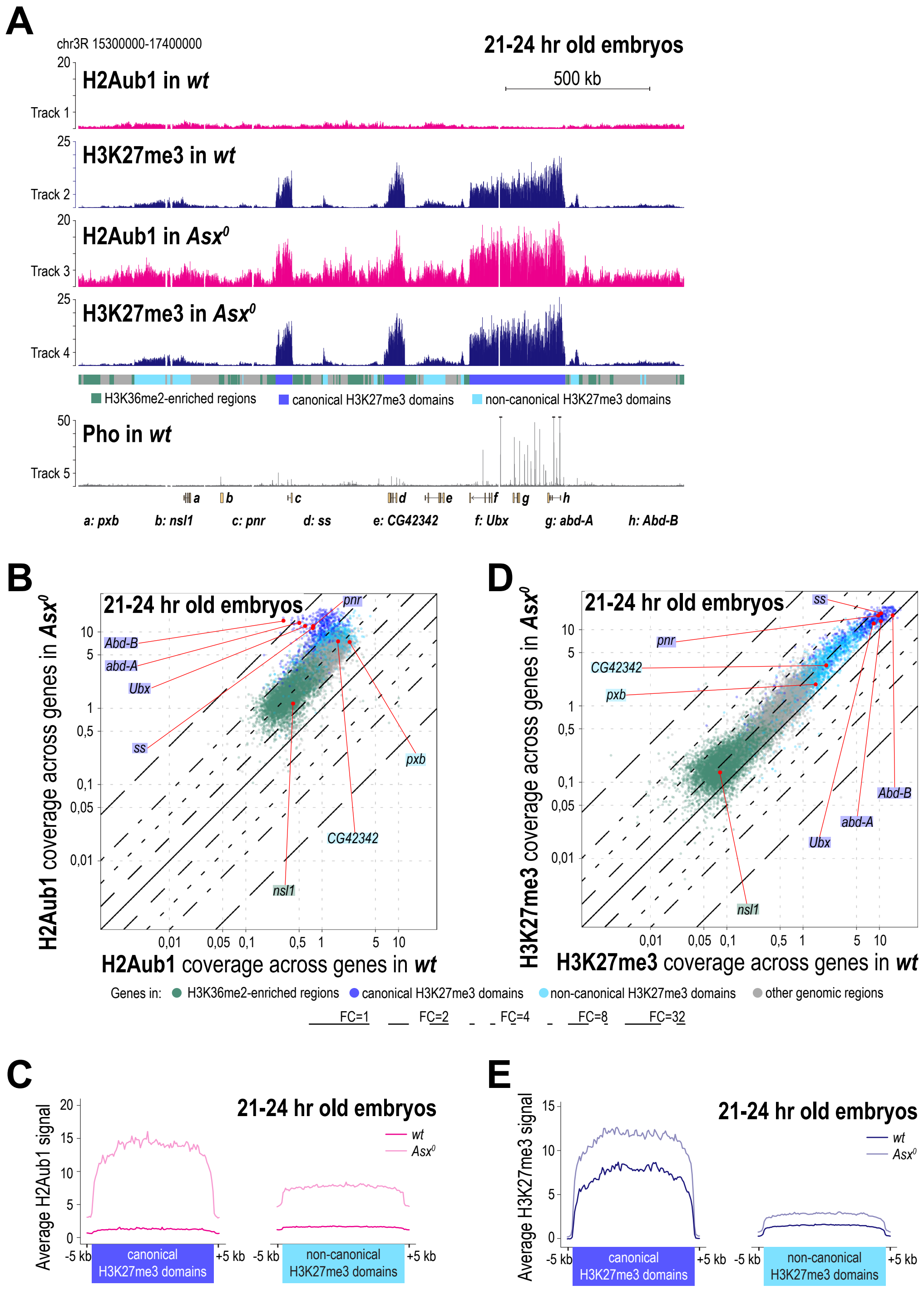
A
chr3R 15300000-17400000
21-24 hr old embryos
500 kb
H2Aub1 in wt
H3K27me3 in wt
H2Aub1 in Asx0
H3K27me3 in Asx0
H3K36me2-enriched regions
canonical H3K27me3 domains
non-canonical H3K27me3 domains
Pho in wt
a: pxb
b: nsl1
c: pnr
d: ss
e: CG42342
f: Ubx
g: abd-A
h: Abd-B
B
H2Aub1 coverage across genes in Asx0
H2Aub1 coverage across genes in wt
D
H3K27me3 coverage across genes in Asx0
H3K27me3 coverage across genes in wt
Genes in: H3K36me2-enriched regions
canonical H3K27me3 domains
non-canonical H3K27me3 domains
other genomic regions
FC=1
FC=2
FC=4
FC=8
FC=32
C
Average H2Aub1 signal
wt
Asx0
-5 kb
+5 kb
canonical H3K27me3 domains
non-canonical H3K27me3 domains
E
Average H3K27me3 signal

Figure S4

Increased H2Aub1 levels in *Asx*^{0} mutant embryos allows formation of regular canonical and non-canonical H3K27me3 domains

(**A**) H2Aub1 and H3K27me3 ChIP-seq profiles at the same chromosomal interval as in **Figures 1A, 2A** and **3A** but comparing 21-24 hrs old wildtype (*wt*) and *Asx*^{0} mutant embryos. Colored bar below track 4 and Pho ChIP-seq profile as references as described in **Figure 1A**.

(**B**) Scatter plots showing H2Aub1 read coverage across gene bodies in 21-24 hrs old wildtype and *Asx*^{0} mutant embryos; compare with H2Aub1 scatter plot in 21-24 hrs old *caly*^{C131S} mutant embryos in **Figure 2B**.

(**C**) Average ChIP-seq profiles for H2Aub1 at H3K27me3 canonical and non-canonical domains in 21-24 hrs old wildtype and *Asx*^{0} mutant embryos (compare with average H2Aub1 profile in 21-24 hrs old *caly*^{C131S} mutant embryos in **Figure 2C**).

(**D**) Same as in (**B**) but for H3K27me3 coverage (compare with scatter plot in 21-24 hrs old *caly*^{C131S} mutant embryos in **Figure 3B**).

(**E**) Same as in (**C**) for H3K27me3 average signal (compare with average H3K27me3 profile in 21-24 hrs old *caly*^{C131S} mutant embryos in **Figure S3B**).

Figure S5

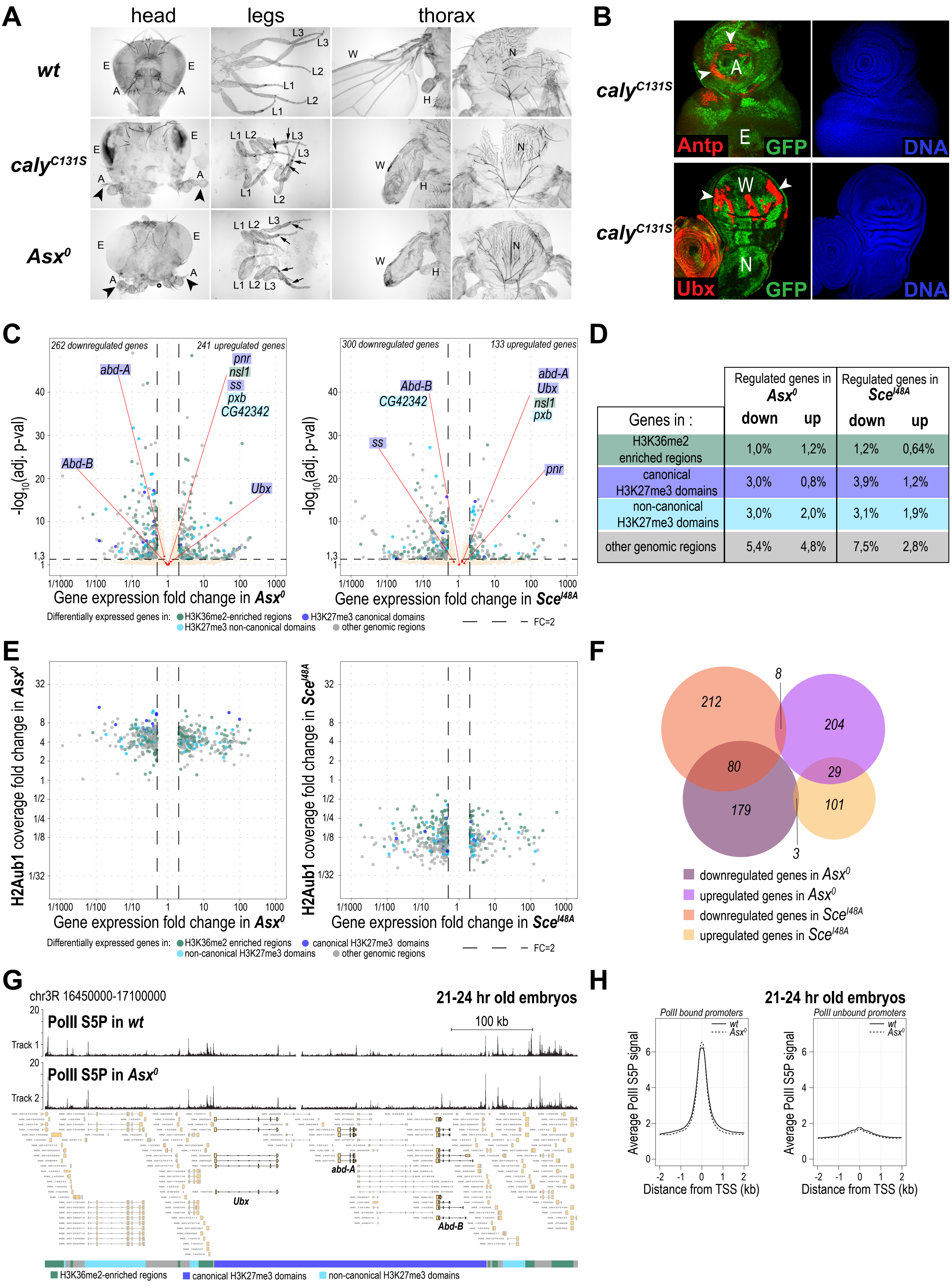
A
head
legs
thorax
wt
calyC131S
Asx0
B
calyC131S
Antp
GFP
DNA
Ubx
C
262 downregulated genes
241 upregulated genes
300 downregulated genes
133 upregulated genes
-log10(adj. p-val)
Gene expression fold change in Asx0
Gene expression fold change in SceI48A
Differentially expressed genes in: H3K36me2-enriched regions H3K27me3 canonical domains H3K27me3 non-canonical domains other genomic regions
FC=2
D
Genes in :
Regulated genes in Asx0 down up
Regulated genes in SceI48A down up
H3K36me2 enriched regions 1,0% 1,2% 1,2% 0,64%
canonical H3K27me3 domains 3,0% 0,8% 3,9% 1,2%
non-canonical H3K27me3 domains 3,0% 2,0% 3,1% 1,9%
other genomic regions 5,4% 4,8% 7,5% 2,8%
E
H2Aub1 coverage fold change in Asx0
H2Aub1 coverage fold change in SceI48A
F
downregulated genes in Asx0
upregulated genes in Asx0
downregulated genes in SceI48A
upregulated genes in SceI48A
G
chr3R 16450000-17100000
21-24 hr old embryos
PolII S5P in wt
PolII S5P in Asx0
100 kb
Track 1
Track 2
Ubx
abd-A
Abd-B
H3K36me2-enriched regions
canonical H3K27me3 domains
non-canonical H3K27me3 domains
H
21-24 hr old embryos
PolII bound promoters
PolII unbound promoters
Average PolII S5P signal
Distance from TSS (kb)

Figure S5

Lack of PR-DUB activity specifically compromises Polycomb repression but not general transcription.

(**A**) PR-DUB mutant cells differentiate to form all epidermal structures in adults and these show tissue-restricted homeotic transformations as the main morphological defect. Preparations of heads with eye (E) and antenna (A), legs (L1 – L3), and thoraces to illustrate wing (W) and haltere (H) or notum (N) structures from adult flies with *yellow*-marked clones of cells that were *wildtype* (*wt*, top row), homozygous for *caly*^{C131S} (middle row) or homozygous for *Asx*^{0} (bottom row). The *yellow*-marked clone tissue is identified by the lighter pigmentation of bristles in this tissue as compared to the darker pigmentation of bristles in the neighbouring wild-type tissue. Clone cells also lacked the *white*$^{+}$ marker which allowed identification of clone tissue in the eye by the absence of red eye pigment. Of note, the animals were in all cases heterozygous for an *M(2)53* mutation which slowed down developmental progression and resulted in a *Minute* phenotype in the animals (Morata & Ripoll, 1975), whereas *y*$^{-}$ clone cells, containing two wild-type *M(2)53*$^{+}$ alleles, grew and proliferated normally, allowing generation of very large *y*$^{-}$-marked clones. In the head, *caly*^{C131S} or *Asx*^{0} mutant tissue in the antenna resulted in partial transformations of the a3 antennal segment and the arista (black arrowheads), with variable expressivity, as previously described (Halachmi *et al*, 2007). But note that *yellow*-marked *caly*^{C131S} or *Asx*^{0} mutant clone tissue in other regions of the head forms regular head structures, and that the arrangement of ommatidia in *caly*^{C131S} or *Asx*^{0} mutant tissue (marked by the absence of red pigment) also appears unperturbed. In the legs, appearance of extra sex combs on the L2 and L3 legs (black arrows) are the only major morphological defect in *caly*^{C131S} or *Asx*^{0} mutants; note that in all three genotypes, the leg tissues consist mostly of clone cells in the individuals shown here. In the thorax, severe transformation of the wing (W) into tissue with characteristics of the haltere (H) is the most prominent morphological defect, whereas in

the notum (N), the structure and bristle pattern formed by *caly*^{C131S} or *Asx*^{0} mutant clone tissue appears indistinguishable from wildtype.

(**B**) Eye-antennal (top) and wing (bottom) imaginal discs with clones of *caly*^{C131S} mutant cells, stained with antibodies against Antp or Ubx (red), as indicated and Hoechst to visualize DNA (blue). The *caly*^{C131S} homozygous mutant clone tissue is marked by the absence of GFP (green). Note that the Antp and Ubx misexpression phenotypes are very similar to those seen in *caly*^{0} or *Asx*^{0} mutants (compare with **Figure 4C**).

(**C**) *Asx*^{0} and *Sce*^{I48A} mutant embryos show no global deregulation of gene transcription. Volcano plots of changes in average gene expression in *Asx*^{0} (left) and *Sce*^{I48A} (right) stage 16 single embryos as compared to single wild-type embryos at the same stage. PolyA+ RNA from single embryos of the three genotypes was quantified by RNA-seq (see Supplemental Experimental Procedures for details). Differentially expressed genes (fold change > 2 and adjusted p-value < 0.05) are represented as dots using the indicated color code. Genes that are not differentially expressed are shown as light orange dots. The genes labelled in Figure1-3 and 7 are marked. We note that *Antp*, *Ubx* and *Abd-B* were not identified as differentially expressed genes in these experiments. This was not unexpected, because *Ubx* and *Antp* are only misexpressed in a small subset of embryonic cells in addition to the cells where they are normally expressed, and *Abd-B* shows a complex alteration of its expression pattern, with both gain and loss of expression (**Figure 4A**). Notwithstanding the notion that cell- or tissue-specific deregulation also of other genes may have escaped detection in RNA-seq analyses in whole embryos, the limited transcriptome alterations in *Asx*^{0} mutants nevertheless argue that the global increase in H2Aub1 coverage across the genome does not cause a gross genome-wide deregulation of gene transcription. Similar arguments apply to the lack of transcriptional deregulation in *Sce*^{I48A} mutant embryos.

(**D**) Percentage of the genes located in each of the different genomic regions identified as differentially expressed genes by RNA-seq.

(**E**) Differentially expressed genes do not fall into a category of genes showing an extreme increase of H2Aub1 levels in *Asx*^{0} mutants (left) or the most severe loss of H2Aub1 in *Sce*^{I48A} mutants (right). Their fold change in gene expression is also not proportional to their H2Aub1 coverage fold-change. Only differentially expressed genes are represented and the color code is as in (**C**).

(**F**) Venn diagram illustrating that the number of genes showing opposite transcriptional defects in *Sce*^{I48A} and *Asx*^{0} mutant embryos is extremely low. The number of genes falling in each category is indicated. A total number of 11813 genes was considered in this analysis.

(**G**) and (**H**) RNA Polymerase II (Pol II) recruitment and Serine 5 phosphorylation of Pol II C-terminal domain at promoters is not affected by the increase in H2Aub1 levels in *Asx*^{0} mutant embryos. (**G**) ChIP-seq profiles for Ser5-phosphorylated Pol II (Pol II S5P) from 21-24 hrs old wildtype (*wt*) or *Asx*^{0} mutant embryos at a genomic window encompassing the *BX-C*. All genes present in this genomic interval are represented on the gene track and the three HOX genes *Ubx*, *abd-A* and *Abd-B* are labelled. (**H**) Average ChIP-seq profile for Pol II S5P, centered on the transcription start site (TSS) of PolII-bound and unbound promoters in wildtype (solid line) and *Asx*^{0} (dotted line) late-stage embryos.

Figure S6

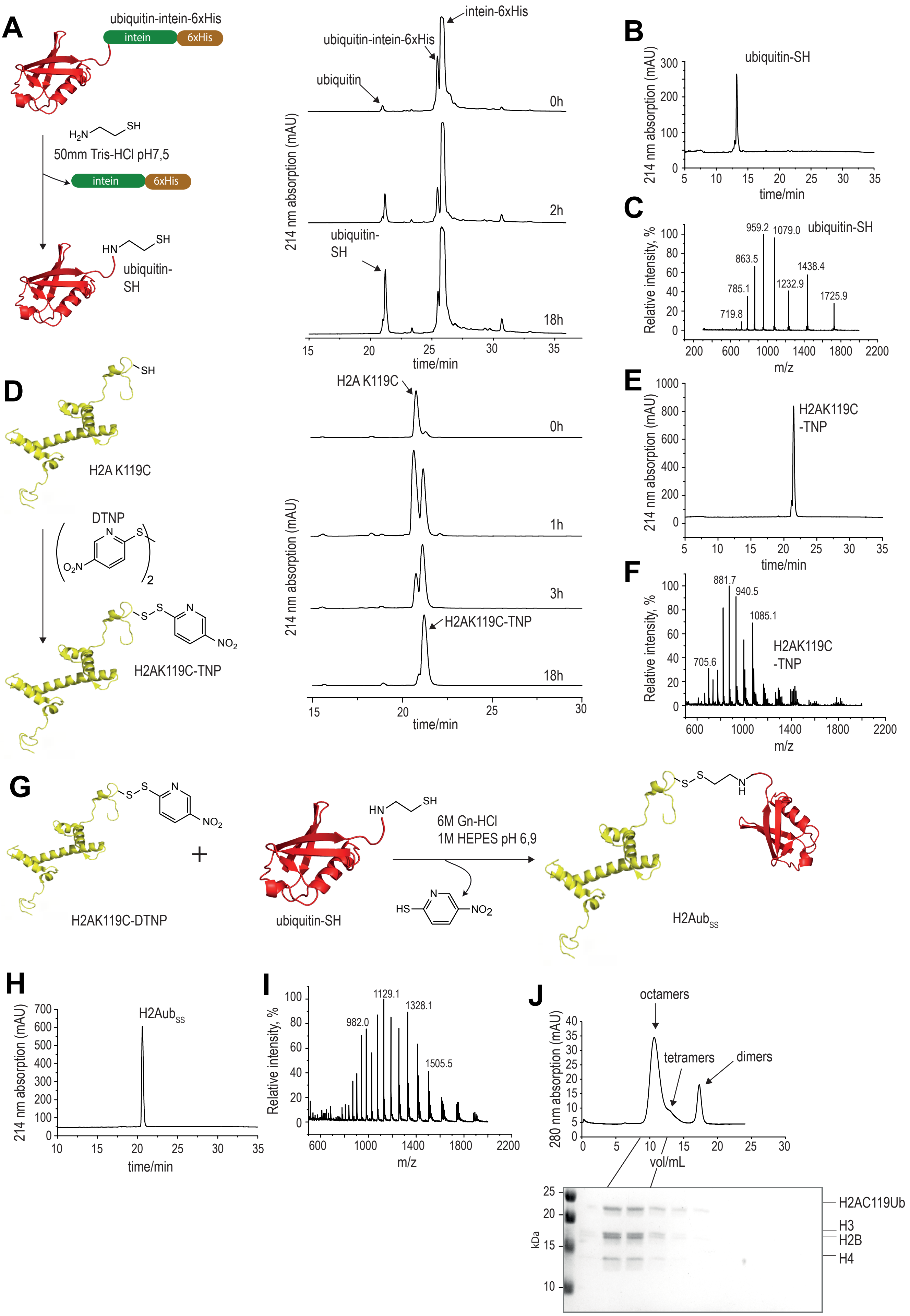

A
ubiquitin-intein-6xHis
intein
6xHis
50mm Tris-HCl pH7,5
ubiquitin-SH
ubiquitin
0h
2h
18h
214 nm absorption (mAU)
time/min
B
ubiquitin-SH
C
959.2
1079.0
863.5
1438.4
785.1
1232.9
1725.9
719.8
Relative intensity, %
m/z
D
H2A K119C
DTNP
H2AK119C-TNP
1h
3h
E
H2AK119C -TNP
F
881.7
940.5
1085.1
705.6
G
H2AK119C-DTNP
6M Gn-HCl
1M HEPES pH 6,9
H2Aub$_{SS}$
H
I
1129.1
1328.1
982.0
1505.5
J
octamers
tetramers
dimers
280 nm absorption (mAU)
vol/mL
kDa
H2AC119Ub
H3
H2B
H4

Figure S6

Synthesis of H2Aub$_{SS}$

(**A**) Production of ubiquitin-SH via Npu intein cleavage using cysteamine. Left: reaction scheme, Right: PR-HPLC analysis of reaction progress over 18h. The large intein-6xHis peak arises from premature intein cleavage and is discarded.

(**B**) Analytical RP-HPLC profile of purified Ubiquitin-SH.

(**C**) ESI-LCMS analysis of ubiquitin-SH (calculated mass: 8615.9 Da, observed mass: 8620.0 Da).

(**D**) Production of H2AK119-TNP via reaction with DTNP intein cleavage using cysteamine. Left: reaction scheme, Right: PR-HPLC analysis of reaction progress over 18h.

(**E**) Analytical RP-HPLC profile of purified H2AK119-TNP.

(**F**) ESI-LCMS analysis of H2AK119-TNP (calculated mass: 14093.40 Da, observed mass: 14094.0 Da).

(**G**) Reaction scheme of the synthesis of H2Aub$_{SS}$.

(**H**) Analytical RP-HPLC profile of purified H2Aub$_{SS}$.

(**I**) ESI-LCMS analysis of H2Aub$_{SS}$ (calculated mass: 22560.24 Da, observed mass: 22560.0 Da).

(**J**) Refolding and purification of H2Aub$_{SS}$ containing histone octamers. Top: Elution profile from size exclusion purification of purified histone octamers. Bottom: SDS-PAGE analysis of elution fractions. Only fractions corresponding to the octamer peak and containing equimolar amounts of all histones were used for chromatin assembly.

Figure S7

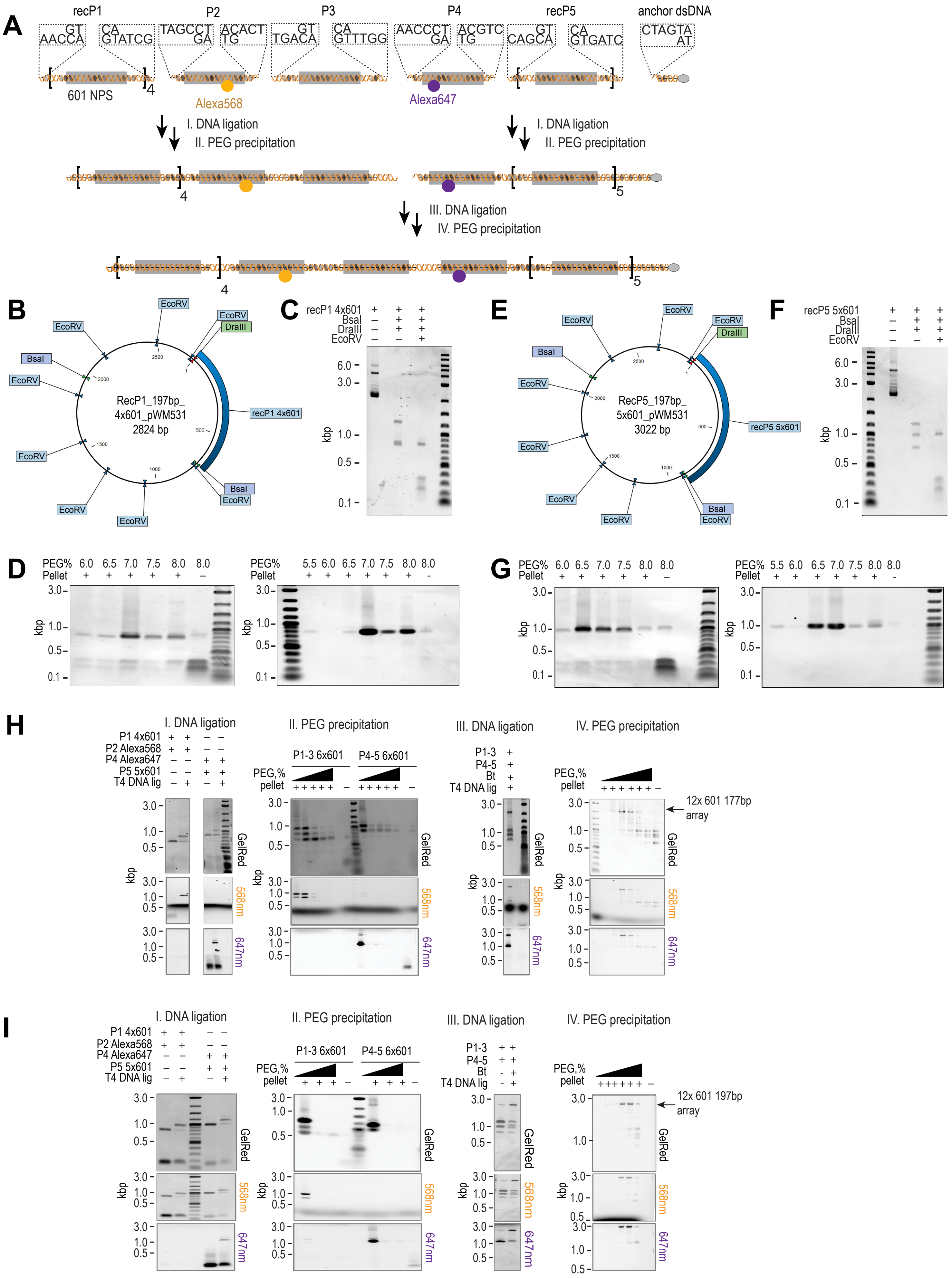
A
recP1
P2
P3
P4
recP5
anchor dsDNA
601 NPS
Alexa568
Alexa647
I. DNA ligation
II. PEG precipitation
III. DNA ligation
IV. PEG precipitation
B
RecP1_197bp_
4x601_pWM531
2824 bp
EcoRV
DraIII
BsaI
recP1 4x601
C
recP1 4x601
BsaI
DraIII
EcoRV
kbp
E
RecP5_197bp_
5x601_pWM531
3022 bp
recP5 5x601
F
recP5 5x601
D
PEG%
Pellet
G
H
P1 4x601
P2 Alexa568
P4 Alexa647
P5 5x601
T4 DNA lig
P1-3 6x601
P4-5 6x601
PEG,%
pellet
P1-3
P4-5
Bt
12x 601 177bp
array
GelRed
568nm
647nm
I
12x 601 197bp
array

Figure S7

Preparation of labeled DNA for smFRET experiments.

(**A**) Scheme for convergent assembly and purification of fluorescently labeled chromatin DNA. Pieces recP1 4x601, P2 1x601 and P3 1x601 are ligated (I. DNA ligation) and the intermediate 6x601 purified by PEG precipitation from the individual pieces (II. PEG precipitation). Simultaneously, P4 1x601, recP5 5x601 and the dsDNA anchor are ligated to produce another 6x601 intermediate (I. DNA ligation, II. PEG precipitation in parallel). The two intermediate 6x601 pieces are ligated to produce the 12x601 chromatin DNA with internal fluorophores (III. DNA ligation) followed by PEG precipitation (IV. PEG precipitation) for final purification.

(**B**) Design of recP1 4x601 in pWM531 (containing 50 bp linker DNA).

(**C**) Excision of piece of recP1 4x601 50 bp linker DNA with non-palindromic overhangs by digestion with BsaI and DraIII followed by plasmid backbone fragmentation by EcoRV.

(**D**) Purification of excised recP1 4x601 by iterative PEG precipitation.

(**E**) Design of recP5 4x601 50 bp linker DNA in pWM531.

(**F**) Excision of piece of recP1 4x601 50 bp linker DNA with non-palindromic overhangs by digestion with BsaI and DraIII followed by plasmid backbone degradation with EcoRV.

(**G**) Purification of recP1 5x601 50bp linker DNA by iterative PEG precipitation.

(**H**) Large scale DNA ligation and PEG precipitations to produce labeled chromatin DNA containing 30 bp linkers.

(**I**) Large scale DNA ligation and PEG precipitations to produce labeled chromatin DNA containing 50 bp linkers.

Figure S8

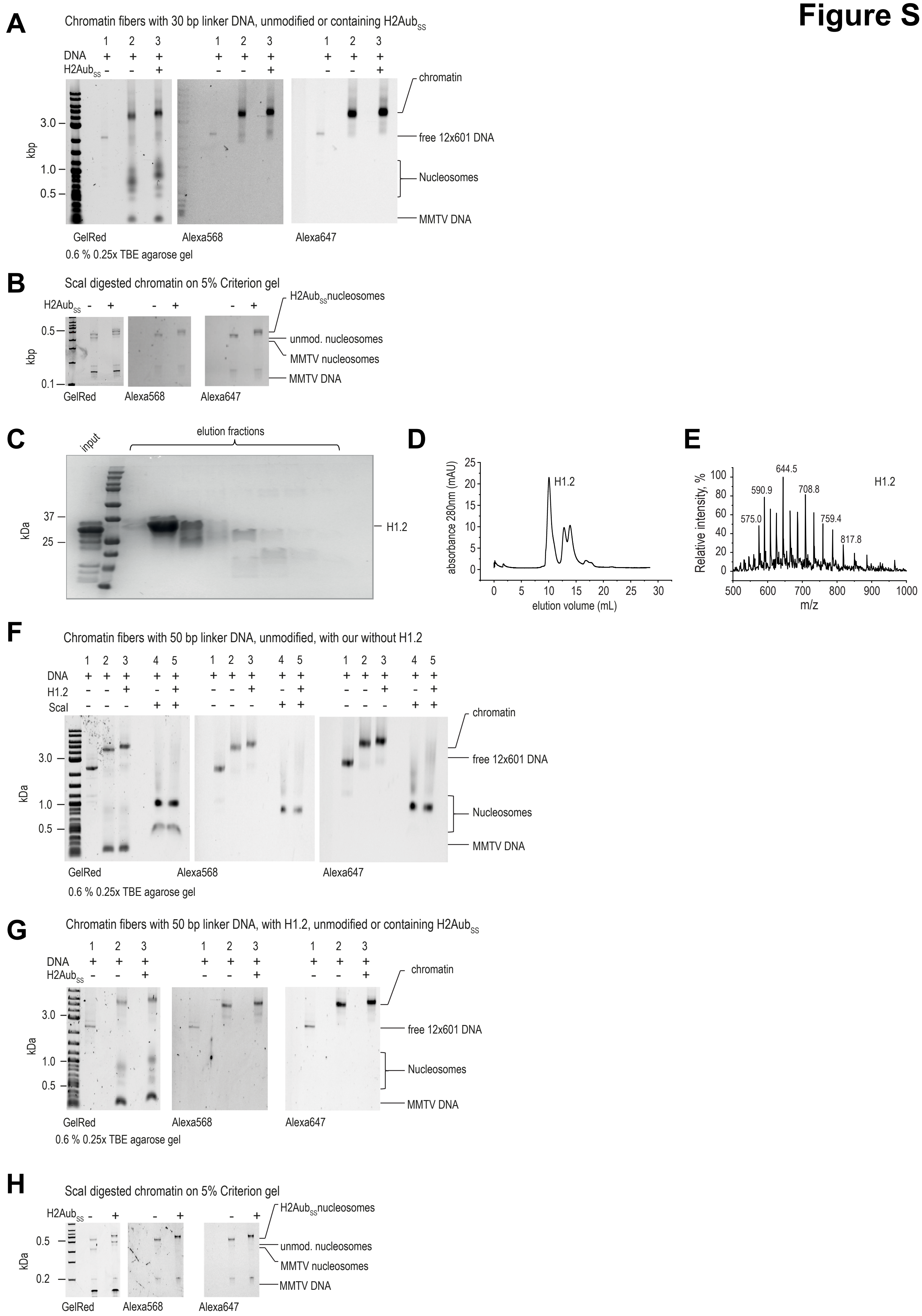
A
Chromatin fibers with 30 bp linker DNA, unmodified or containing H2Aub$_{SS}$
DNA
H2Aub$_{SS}$
chromatin
free 12x601 DNA
Nucleosomes
MMTV DNA
kbp
3.0
1.0
0.5
GelRed
Alexa568
Alexa647
0.6 % 0.25x TBE agarose gel
B
ScaI digested chromatin on 5% Criterion gel
H2Aub$_{SS}$ nucleosomes
unmod. nucleosomes
MMTV nucleosomes
MMTV DNA
0.5
0.1
C
input
elution fractions
kDa
37
25
H1.2
D
absorbance 280nm (mAU)
elution volume (mL)
H1.2
E
Relative intensity, %
m/z
H1.2
575.0
590.9
644.5
708.8
759.4
817.8
F
Chromatin fibers with 50 bp linker DNA, unmodified, with our without H1.2
DNA
H1.2
ScaI
chromatin
free 12x601 DNA
Nucleosomes
MMTV DNA
kDa
GelRed
Alexa568
Alexa647
0.6 % 0.25x TBE agarose gel
G
Chromatin fibers with 50 bp linker DNA, with H1.2, unmodified or containing H2Aub$_{SS}$
DNA
H2Aub$_{SS}$
chromatin
free 12x601 DNA
Nucleosomes
MMTV DNA
GelRed
Alexa568
Alexa647
0.6 % 0.25x TBE agarose gel
H
ScaI digested chromatin on 5% Criterion gel
H2Aub$_{SS}$ nucleosomes
unmod. nucleosomes
MMTV nucleosomes
MMTV DNA
0.5
0.2
GelRed
Alexa568
Alexa647

Figure S8

Assembly of chromatin fibers containing H2Aub$_{SS}$ and H1.2

(**A**) Analysis of chromatin formation on fluorescently labeled DNA (30 bp linker) by agarose gel electrophoresis, stained with GelRed or imaged at wavelengths appropriate for the indicated fluorescent dyes. Lane 1: Free labeled array DNA. Lane 2: Assembled chromatin arrays with unmodified histone octamers. To avoid overloading array DNA with histone octamers, low-affinity buffer DNA (mouse mammary tumor virus (MMTV) sequence) is added, resulting in the formation of a small amount of buffer nucleosomes (MMTV nucleosomes). Lane 3: Assembled chromatin arrays with H2Aub$_{SS}$ histone octamers.

(**B**) Digestion of indicated chromatin fibers with the restriction enzyme ScaI, liberating mononucleosomes. The absence of higher-order aggregates or significant amounts of free DNA demonstrates the saturation of chromatin arrays.

(**C**) Ion exchange purification of H1.2. Concentrated elution fractions showing the least amount of degradation are used for further purification.

(**D**) Size exclusion chromatography purification of H1.2.

(**E**) ESI-LCMS analysis of purified H1.2 (calculated mass: 21233.56 Da, measured mass: 21236.0 Da).

(**F**) Agarose gel electrophoresis analysis of chromatin formation on fluorescently labeled DNA (50 bp linker) (Lane 1), with our without H1.2 (Lanes 2-3) and ScaI digestion (Lanes 4-5).

(**G**) Agarose gel electrophoresis analysis of chromatin formation on fluorescently labeled DNA (50 bp linker) (Lane 1), with H1.2 and with our without H2Aub$_{SS}$ (Lanes 2-3).

(**H**) ScaI digestion analysis of chromatin assemblies on fluorescently labeled DNA (50 bp linker) with H1.2 and with our without H2Aub$_{SS}$.

Figure S9

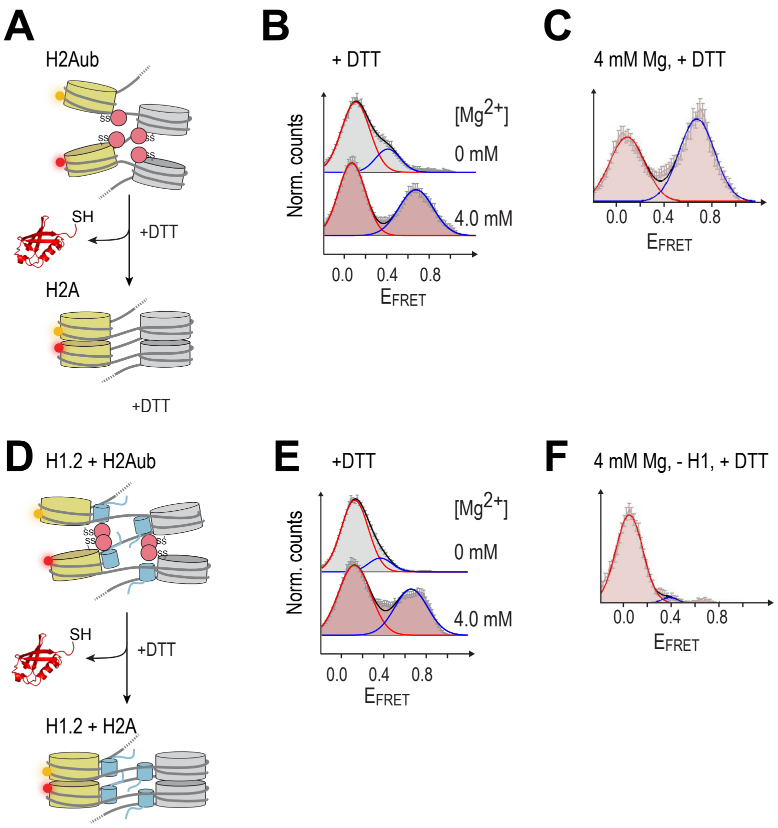
A
H2Aub
SH
+DTT
H2A
+DTT
B
+ DTT
[Mg2+]
0 mM
4.0 mM
Norm. counts
0.0 0.4 0.8
EFRET
C
4 mM Mg, + DTT
0.0 0.4 0.8
EFRET
D
H1.2 + H2Aub
SH
+DTT
H1.2 + H2A
E
+DTT
[Mg2+]
0 mM
4.0 mM
Norm. counts
0.0 0.4 0.8
EFRET
F
4 mM Mg, - H1, + DTT
0.0 0.4 0.8
EFRET

Figure S9

Chemically deubiquitinated chromatin exhibits nucleosome stacking.

(**A**) H2Aub$_{SS}$ containing chromatin fibers (30 bp linkers) are treated with 100 mM DTT to remove the ubiquitin moiety via reduction of the disulfide bond.

(**B**) smFRET histograms for H2Aub$_{SS}$ containing chromatin fibers with 30 bp linker DNA, at the indicated Mg^{2+} concentrations after 100 mM DTT treatment.

(**C**) Addition of DTT does not influence chromatin compaction: smFRET histogram for unmodified chromatin fibers with 30 bp linker DNA, at 4 mM Mg^{2+} in the presence of 100 mM DTT.

(**D**) H2Aub$_{SS}$ containing chromatin fibers (50 bp linkers, containing H1.2) are deubiquitinated with 100 mM DTT.

(**E**) smFRET histograms for H2Aub$_{SS}$ containing chromatin fibers with 50 bp linker DNA and H1.2, at the indicated Mg^{2+} concentrations after 100 mM DTT treatment.

(**F**) Chromatin fibers with 50 bp linkers cannot compact in the absence of H1.2 when exposed to 100 mM DTT: smFRET histogram for unmodified chromatin fibers with 50 bp linker DNA, at 4 mM Mg^{2+} in the presence of 100 mM DTT.

Figure S10

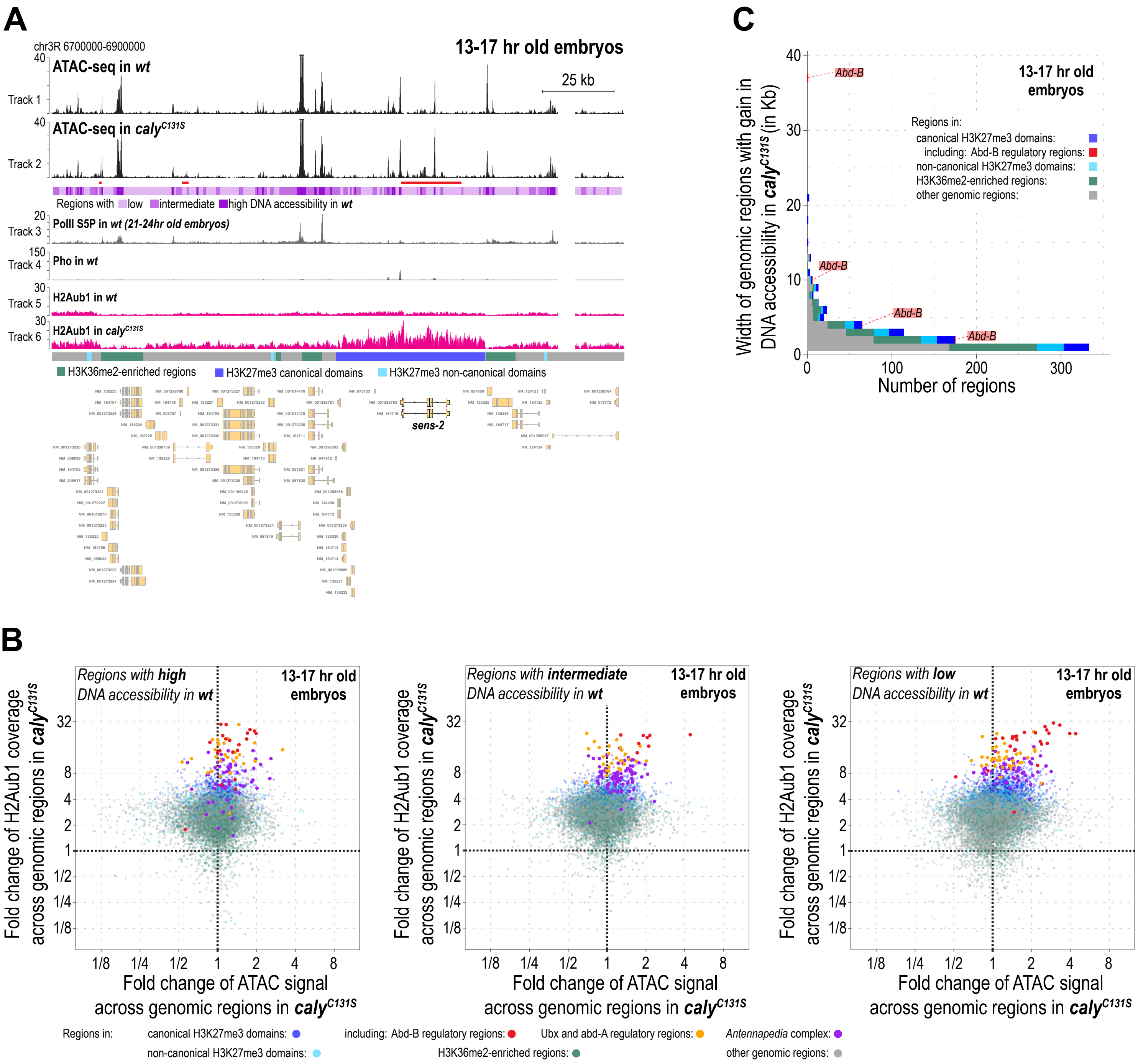

A
chr3R 6700000-6900000
13-17 hr old embryos
25 kb
Track 1
ATAC-seq in wt
Track 2
ATAC-seq in caly^C131S
Regions with low intermediate high DNA accessibility in wt
Track 3
PolII S5P in wt (21-24hr old embryos)
Track 4
Pho in wt
Track 5
H2Aub1 in wt
Track 6
H2Aub1 in caly^C131S
H3K36me2-enriched regions
H3K27me3 canonical domains
H3K27me3 non-canonical domains
sens-2
C
13-17 hr old embryos
Regions in:
canonical H3K27me3 domains:
including: Abd-B regulatory regions:
non-canonical H3K27me3 domains:
H3K36me2-enriched regions:
other genomic regions:
Abd-B
Width of genomic regions with gain in DNA accessibility in caly^C131S (in Kb)
Number of regions
B
Regions with high DNA accessibility in wt
Regions with intermediate DNA accessibility in wt
Regions with low DNA accessibility in wt
13-17 hr old embryos
Fold change of H2Aub1 coverage across genomic regions in caly^C131S
Fold change of ATAC signal across genomic regions in caly^C131S
Regions in:
canonical H3K27me3 domains:
including: Abd-B regulatory regions:
Ubx and abd-A regulatory regions:
Antennapedia complex:
non-canonical H3K27me3 domains:
H3K36me2-enriched regions:
other genomic regions:

Figure S10

Excessive H2Aub1 levels lead to chromatin opening *in vivo*.

(**A**) ATAC-seq profiles from 13-17 hrs old wildtype (*wt*) and *caly*^{C131S} mutant embryos at a genomic window encompassing the *sens-2* gene, which is located in a canonical H3K27me3 domain. Red bars below Track 2 underline regions with significant gain in DNA accessibility in *caly*^{C131S} mutants (see also **Figure 5H-I**, and Supplemental Experimental Procedures). RNA Pol II S5P, Pho and H2Aub1 profiles are shown as reference. Pho-bound PREs and RNA Pol II S5P-bound promoters show high DNA accessibility in both genotypes, but note the increased accessibility at *sens-2* between peaks in *caly*^{C131S} mutants (red bar below track 2). Also note the high H2Aub1 level increase in *caly*^{C131S} mutants across this same region.

(**B**) Scatter plots as in **Figure 5I**, but representing fold changes of ATAC-seq signal and H2Aub1 read coverage in 13-17 hrs old *caly*^{C131S} mutant embryos separately for genomic regions with high (left), intermediate (middle) and low (right) DNA accessibility.

(**C**) Histogram showing the distribution of the length of genomic regions exhibiting a significant increase in DNA accessibility in *caly*^{C131S} embryos. The *Abd-B* gene locus (in red) is among the regions in the genome, where the excess of H2Aub1 has the strongest effect on the opening of chromatin. Each region is colored based on the type of chromatin with which it overlaps.

Figure S11

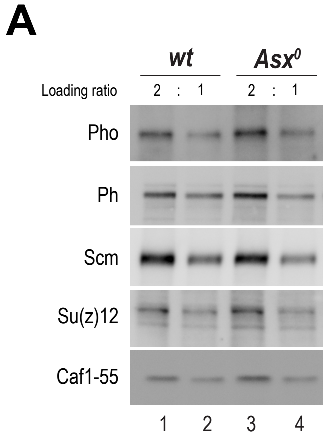
A
wt
Asx0
Loading ratio
2 : 1
2 : 1
Pho
Ph
Scm
Su(z)12
Caf1-55
1 2 3 4

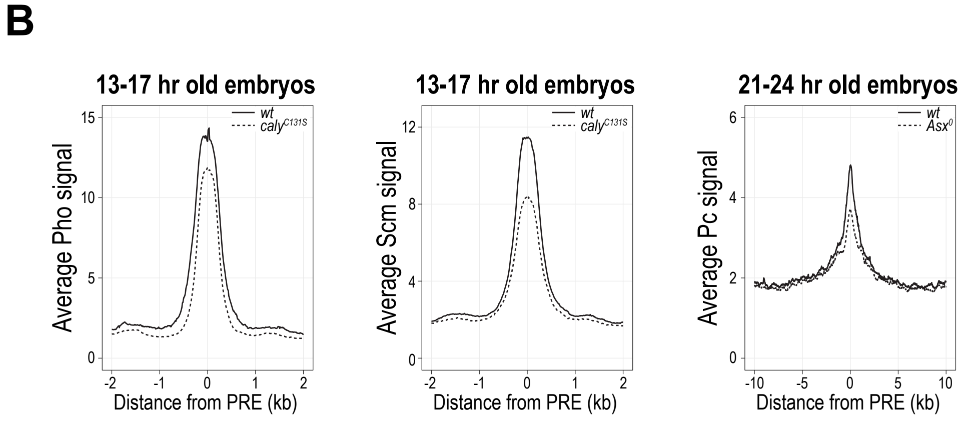
B
13-17 hr old embryos
Average Pho signal
Distance from PRE (kb)
wt
calyC131S
13-17 hr old embryos
Average Scm signal
Distance from PRE (kb)
wt
calyC131S
21-24 hr old embryos
Average Pc signal
Distance from PRE (kb)
wt
Asx0

Figure S11

Recruitment of PcG proteins at PREs is unimpaired in PR-DUB mutant embryos

(**A**) Western blot analysis on serial dilutions of total nuclear extracts from 21-24 hrs old embryos shows that the levels of Pho, Ph, Scm and Su(z)12 in Asx^{0} mutant embryos are comparable to wildtype (*wt*).

(**B**) PRE-centered average ChIP-seq profiles for Pho, Scm and Pc, in wildtype (solid line) and PR-DUB mutant (dotted line) embryos. Pho and Scm ChIP-seq meta-analysis were performed on 13-17 hrs old wildtype and $caly^{C131S}$ mutant embryos. Pc ChIP-seq meta-analysis was performed on 21-24 hrs old wildtype and Asx^{0} mutant embryos. Coordinates of PREs correspond to the centers of 365 Pho peaks that are not more than 150 bp away from the center of a Scm peak and that locate in a canonical H3K27me3 domain.

Table S1

Mutant alleles and genotypes of animals used in the different figures

The following **mutant alleles** were used in this study:

caly^{C131S}: generated in this study, see Material and Methods for details.

caly^{2}: null allele (Scheuermann *et al*, 2010), referred to as *caly*^{0} in this manuscript.

Sce^{KO}: null allele (Gutiérrez *et al*, 2012), referred to as *Sce*^{0} in this manuscript.

Asx^{22P4}: null allele (Scheuermann *et al*, 2010) referred to as *Asx*^{0} in this manuscript.

Pc^{XT109}: null allele (Franke *et al*, 1995) referred to as Pc^{0} in this manuscript.

M(2)53: mutation which slows down developmental progression and results in a *Minute* phenotype (Morata & Ripoll, 1975).

Genotypes of animals used in the different figures

Figure 1:

wt: *Oregon-R*

Sce^{I48A}: obtained as GFP-negative progeny in crosses from mothers and fathers of the following genotype:

w; Sce^{I48A} *(VK37) >Sce*$^{+}$*> (J27) / Sce*^{I48A}*(VK37); nos-Gal4VP16 F82B cu sr Sce*^{0} */ UAS-FLP(VK33) F82B cu sr Sce*^{0} (see (Pengelly *et al*, 2015) for more details*)*

Figure 2:

wt: *Oregon-R*

caly^{C131S}: obtained as GFP-negative progeny from mothers and fathers of the following genotype:

w; caly^{C131S} *nos-flp-nos (VK18) / caly*^{C131S}*; >caly*$^{+}$*> (VK33) nos-Gal4VP16 / +*

Figure 3:

wt: *Oregon-R*

Sce^{I48A}: obtained as GFP-negative progeny from mothers and fathers of the following genotype:

w; Sce^{I48A} *(VK37) >Sce*$^{+}$*> (J27) / Sce*^{I48A}*(VK37); nos-Gal4VP16 F82B cu sr Sce*^{0} */ UAS-FLP(VK33) F82B cu sr Sce*^{0} (see (Pengelly *et al*, 2015) for more details*)*

caly^{C131S}: obtained as GFP-negative progeny from mothers and fathers of the following genotype:

w; caly^{C131S} nos-flp-nos (VK18) / caly^{C131S}; >caly^{+}> (VK33) nos-Gal4VP16 / +

Figure 4A:

wt: *Oregon-R*

caly^{C131S}: obtained as GFP-negative progeny from mothers and fathers of the following genotype:

w; caly^{C131S} nos-flp-nos (VK18) / caly^{C131S}; >caly^{+}> (VK33) nos-Gal4VP16 / +

caly^{0}: obtained as GFP-negative progeny from heat-shocked mothers that were *yw hs-Flp122 / w; F40 F42D y^{+} caly^{0} / F42D ovo^{D1} w^{+}* and were crossed to *w / Y; F40 F42D y^{+} caly^{0} / CyO ubi-GFP* fathers.

Asx^{0}: obtained as GFP-negative progeny from mothers and fathers of the following genotype:

w; F40 F42D y^{+} Asx^{0} / Cyo twi-Gal4 UAS-GFP

Figure 4B:

Sce^{I48A}: obtained as GFP-negative progeny from mothers and fathers of the following genotype:

w; Sce^{I48A} (VK37) >Sce^{+}> (J27) / Sce^{I48A}(VK37); nos-Gal4VP16 F82B cu sr Sce^{0} / UAS-Flp(VK33) F82B cu sr Sce^{0} (see (Pengelly *et al*, 2015) for more details*)*

Asx^{0} Sce^{I48A}: obtained as GFP-negative progeny from heat-shocked mothers that were: *w hs-Flp122 / w; Sce^{I48A} (VK37) F42D y^{+} Asx^{0} / Cyo twi-Gal4 UAS-GFP; F82B Sce^{0} / F82B ovo^{D1} w^{+}* and were crossed to *w / Y; Sce^{I48A} (VK37) F42D y^{+} Asx^{0} / Cyo twi-Gal4 UAS-GFP; F82B Sce^{0} / TM6B* fathers. To exclude the embryos which received the TM6B balancer chromosome carrying a wildtype *Sce^{+}* allele, the collected GFP-negative embryos were first stained with H2Aub1 antibodies and those lacking H2Aub1 signal (i.e. the *w hs-Flp122 / w; Sce^{I48A} (VK37) F42D y^{+} Asx^{0} / Sce^{I48A} (VK37) F42D y^{+} Asx^{0}; F82B Sce^{0} / F82B Sce^{0}* embryos) were then stained for Abd-B.

Figure 4C:

Asx^{0}: GFP-negative clones of *Asx^{0}* homozygous cells were induced by Flp expression in animals of the following genotype:

yw hs-Flp122; F40 F42D y^{+}Asx^{0} / F42D hs-nGFP

Asx^{0} Sce^{I48A}: GFP-negative clones of *Asx^{0}* homozygous cells were induced by Flp expression in animals of the following genotype:

yw hs-Flp122; Sce^{I48A}(VK37) F42D Asx^{0} / F42D hs-nGFP; F82B Sce^{0}/ F82B Sce^{0}

caly^{0}: GFP-negative clones of *caly^{0}* homozygous cells were induced by Flp expression in animals of the following genotype:

yw hs-Flp122; F40 F42D y^{+} caly^{0} / F42D hs-nGFP

caly^{0} Sce^{I48A}: GFP-negative clones of *caly^{0}* homozygous cells were induced by Flp expression in animals of the following genotype: *yw hs-Flp122; Sce^{I48A}(VK37) F42D caly^{0} / F42D hs-nGFP; F82B Sce^{0}/ F82B Sce^{0}*

Figure 5 H-I:

wt: *Oregon-R*

$caly^{C131S}$: obtained as GFP-negative progeny from mothers and fathers of the following genotype:

w; $caly^{C131S}$ nos-flp-nos (VK18) / $caly^{C131S}$; >$caly^{+}$> (VK33) nos-Gal4VP16 / +

Figure 6A

wt: *Oregon-R*

$caly^{C131S}$: obtained as GFP-negative progeny from mothers and fathers of the following genotype:

w; $caly^{C131S}$ nos-flp-nos (VK18) / $caly^{C131S}$; >$caly^{+}$> (VK33) nos-Gal4VP16 / +

Asx^0: obtained as GFP-negative progeny from mothers and fathers of the following genotype: *w; F40 F42D y^+ Asx^0 / Cyo twi-Gal4 UAS-GFP*

Figure 6B:

wt: *Oregon-R*

Pc^0 / +: obtained as GFP-positive progeny from: *Pc^0 F2A / TM3 twi-GFP* and identified as *Pc^0* heterozygotes by the intensity of the GFP signal.

Asx^0: obtained as GFP-negative progeny from mothers and fathers of the following genotype:

w; F40 F42D y^+ Asx^0 / Cyo twi-Gal4 UAS-GFP

Asx^0; Pc^0 / +: obtained as the progeny of a cross between mothers that were *w; F42D y+ Asx^0 / CyO ubi-GFP; Pc^0 F2A / TM6B* and fathers that were *w; F42D y+ Asx^0 / CyO ubi-GFP*. The GFP-negative progeny fell into two distinct classes: in *class I* embryos, the embryonic cuticle and HOX gene expression patterns were indistinguishable from *Asx^0/Asx^0* embryos, these were the presumed *w; F42D y+ Asx^0 / F42D y+ Asx^0; TM6B/* + animals; *class II* embryos all showed enhanced misexpression of HOX genes *Antp*, *Ubx* and *Abd-B*, and more severe homeotic transformations in the embryonic cuticle, as illustrated in Figure 6B, these were the presumed *w; F42D y+ Asx^0 / F42D y+ Asx^0; Pc^0 F2A/* + animals.

Figure S1:

wt: *Oregon-R*

Figure S2B:

$caly^{C131S}$: GFP-negative clones of *$caly^{C131S}$* homozygous cells were induced by Flp expression in animals of the following genotype: *w hs-Flp122; F42D y^+ $caly^{C131S}$ / F42D hs-nGFP*

$caly^0$: GFP-negative clones of *$caly^0$* homozygous cells were induced by Flp expression in animals of the following genotype: *w hs-Flp122; F40 F42D y^+ $caly^0$ / F42D hs-nGFP*

Figure S2E:

wt: *Oregon-R*

Sce^{I48A}: obtained as GFP-negative progeny from mothers and fathers of the following genotype:

w; Sce^{I48A} (VK37) >Sce^{+}> (J27) / Sce^{I48A}(VK37); nos-Gal4VP16 F82B cu sr Sce^{0} / UAS-Flp(VK33) F82B cu sr Sce^{0} (see (Pengelly *et al*, 2015) for more details*)*

caly^{C131S}: obtained as GFP negative progeny from mothers and fathers of the following genotype:

w; caly^{C131S} nos-flp-nos (VK18) / caly^{C131S}; >caly^{+}> (VK33) nos-Gal4VP16 / +

Asx^{0}: obtained as GFP negative progeny from mothers and fathers of the following genotype:

w; F40 F42D y^{+} Asx^{0} / Cyo twi-Gal4 UAS-GFP

Figure S3:

wt: *Oregon-R*

Sce^{I48A}: obtained as GFP-negative progeny from mothers and fathers of the following genotype:

w; Sce^{I48A} (VK37) >Sce^{+}> (J27) / Sce^{I48A}(VK37); nos-Gal4VP16 F82B cu sr Sce^{0} / UAS-FLP(VK33) F82B cu sr Sce^{0} (see (Pengelly *et al*, 2015) for more details*)*

caly^{C131S}: obtained as GFP-negative progeny from mothers and fathers of the following genotype:

w; caly^{C131S} nos-flp-nos (VK18) / caly^{C131S}; >caly^{+}> (VK33) nos-Gal4VP16 / +

Figure S4:

wt: *Oregon-R*

Asx^{0}: obtained as GFP negative progeny from mothers and fathers of the following genotype:

w; F40 F42D y^{+} Asx^{0} / Cyo twi-Gal4 UAS-GFP

Figure S5A:

wt: clones of *wt* (+/+) cells, marked by absence of *y^{+}*, were induced by Flp expression in animals of the following genotype:

yw hs-Flp122 / Y; F42D +/ F42D y^{+} M(2)53 DsRed

caly^{C131S}: clones of *caly^{C131S}* homozygous cells, marked by absence of *y^{+}*, were induced by Flp expression in animals of the following genotype:

yw hs-Flp122 / Y; F42D caly^{C131S}/ F42D y^{+} M(2)53 DsRed

Asx^{0}: clones of *Asx^{0}* homozygous cells, marked by absence of *y^{+}*, were induced by Flp expression in animals of the following genotype:

yw hs-Flp122 / Y; F42D Asx^{0}/ F42D y^{+} M(2)53 DsRed

Figure S5B:

caly^{C131S}: GFP-negative clones of *caly^{C131S}* homozygous cells were induced by Flp expression in animals of the following genotype:

yw hs-Flp122; F42D caly^{C131S} / F42D hs-nGFP

Figure S5C-H:

wt: *Oregon-R*

Asx^{0}: obtained as GFP negative progeny from mothers and fathers of the following genotype:

w; F40 F42D y^{+} Asx^{0} / Cyo twi-Gal4 UAS-GFP

Sce^{I48A}: obtained as GFP-negative progeny from mothers and fathers of the following genotype:

w; Sce^{I48A} (VK37) >Sce^{+}> (J27) / Sce^{I48A}(VK37); nos-Gal4VP16 F82B cu sr Sce^{0} / UAS-Flp(VK33) F82B cu sr Sce^{0} (see (Pengelly *et al*, 2015) for more details*)*

Figure S10

wt: *Oregon-R*

caly^{C131S}: obtained as GFP negative progeny from mothers and fathers of the following genotype:

w; caly^{C131S} nos-flp-nos (VK18) / caly^{C131S}; >caly^{+}> (VK33) nos-Gal4VP16 / +

Figure S11

wt: *Oregon-R*

Asx^{0}: obtained as GFP negative progeny from mothers and fathers of the following genotype:

w; F40 F42D y^{+} Asx^{0} / Cyo twi-Gal4 UAS-GFP

caly^{C131S}: obtained as GFP negative progeny from mothers and fathers of the following genotype:

w; caly^{C131S} nos-flp-nos (VK18) / caly^{C131S}; >caly^{+}> (VK33) nos-Gal4VP16 / +

Table S2

Gaussian fit results from the indicated smFRET experiments, as well as numbers of independent experiments.

DNA	Sample	Mg2+ (mM)	A1	c1	d1	A2	c2	d2	n indep. exp.	n traces
177 bp	unmod.	0	0.55	0.05	0.13	0.45	0.34	0.13	2	368
177 bp	unmod.	4	0.4	0.08	0.14	0.6	0.66	0.14	2	348
177 bp	H2A119Ub	0	0.97	0.07	0.13	0.03	0.53	0.13	4	340
177 bp	H2A119Ub	4	0.97	0.09	0.14	0.03	0.6	0.16	4	361
177 bp	H2A119Ub + 100 mM DTT	0	0.75	0.09	0.13	0.25	0.39	0.11	2	296
177 bp	H2A119Ub + 100 mM DTT	4	0.61	0.05	0.12	0.39	0.65	0.16	3	411
177 bp	unmod + 100 mM DTT	4	0.44	0.09	0.15	0.56	0.67	0.15	1	265
197 bp	unmod.	0	0.97	0.09	0.15	0.03	0.44	0.08	3	967
197 bp	unmod.	4	0.75	0.06	0.15	0.25	0.36	0.16	3	1477
197 bp	unmod. + H1	0	0.74	0.15	0.16	0.26	0.42	0.11	3	732
197 bp	unmod. + H1	4	0.55	0.12	0.16	0.45	0.63	0.16	3	658
197 bp	H2A119Ub + H1	0	0.99	0.09	0.12	0.01	0.42	0.04	3	379
197 bp	H2A119Ub + H1	4	0.98	0.11	0.13	0.02	0.56	0.08	3	438
197 bp	H2A119Ub + H1 + 100 mM DTT	0	0.84	0.12	0.13	0.16	0.36	0.11	2	483
197 bp	H2A119Ub + H1 + 100 mM DTT	4	0.6	0.11	0.15	0.4	0.66	0.16	3	667
197 bp	H2A119Ub	0	0.97	0.06	0.11	0.03	0.83	0.08	1	45
197 bp	H2A119Ub	4	1	0.04	0.11	0	0.75	0.01	1	59
197 bp	H2A119Ub + DTT	4	0.94	0.04	0.11	0.06	0.38	0.07	1	62

Table S3

Antibodies used in this study

Specificity	Source / Reference
H2AK119ub	Cell Signaling (D27C4)
H3	Abcam (ab 1791)
H3K27me3	Cell Signaling (C36B11)
Caf1	Gambetta et al. 2009
E(z)	Gambetta et al. 2009
Pc	Papp and Müller 2006
Ph	Oktaba et al. 2008
Pho	Papp and Müller 2006
Scm	Gambetta et al. 2009
Su(z)12	Müller et al. 2002
Abd-B	DSHB (1A2E9)
Antp	DSHB (8C11)
Ubx	DSHB (FP3.38)

Supplemental References

Bonnet J, Lindeboom RGH, Pokrovsky D, Stricker G, Çelik MH, Rupp RAW, Gagneur J, Vermeulen M, Imhof A & Müller J (2019) Quantification of Proteins and Histone Marks in *Drosophila* Embryos Reveals Stoichiometric Relationships Impacting Chromatin Regulation. *Dev. Cell* **51:** 632–644.e6

Chatterjee C, McGinty RK, Fierz B & Muir TW (2010) Disulfide-directed histone ubiquitylation reveals plasticity in hDot1L activation. *Nat. Chem. Biol.* **6:** 267–269

Debelouchina GT, Gerecht K & Muir TW (2017) Ubiquitin utilizes an acidic surface patch to alter chromatin structure. *Nat. Chem. Biol.* **13:** 105–110

Dobin A, Davis CA, Schlesinger F, Drenkow J, Zaleski C, Jha S, Batut P, Chaisson M & Gingeras TR (2013) STAR: ultrafast universal RNA-seq aligner. *Bioinformatics* **29:** 15–21

Dos Santos G, Schroeder AJ, Goodman JL, Strelets VB, Crosby MA, Thurmond J, Emmert DB, Gelbart WM, Brown NH, Kaufman T, Werner-Washburne M, Cripps R, Broll K, Gramates LS, Falls K, Matthews BB, Russo S, Zhou P, Zytkovicz M, Adryan B, et al (2015) FlyBase: Introduction of the *Drosophila melanogaster* Release 6 reference genome assembly and large-scale migration of genome annotations. *Nucleic Acids Res.* **43:** D690–D697

Fierz B, Chatterjee C, McGinty RK, Bar-Dagan M, Raleigh DP & Muir TW (2011) Histone H2B ubiquitylation disrupts local and higher-order chromatin compaction. *Nat. Chem. Biol.* **7:** 113–119

Finogenova K, Bonnet J, Poepsel S, Schäfer IB, Finkl K, Schmid K, Litz C, Strauss M, Benda C & Müller J (2020) Structural basis for PRC2 decoding of active histone methylation marks H3K36me2/3. *eLife* **9:** e61964

Franke A, Messmer S & Paro R (1995) Mapping functional domains of the Polycomb protein of *Drosophila melanogaster*. *Chromosome Res.* **3:** 351–360

Gambetta MC & Müller J (2014) O-GlcNAcylation Prevents Aggregation of the Polycomb Group Repressor Polyhomeotic. *Dev. Cell* **31:** 629–639

Gutiérrez L, Oktaba K, Scheuermann JC, Gambetta MC, Ly-Hartig N & Müller J (2012) The role of the histone H2A ubiquitinase Sce in Polycomb repression. *Development* **139:** 117–127

Halachmi N, Schulze KL, Inbal A & Salzberg A (2007) *Additional sex combs* Affects Antennal Development by Means of Spatially Restricted Repression of *Antp* and *wg*. **236:** 2118–2130

Kaushal A, Mohana G, Dorier J, Özdemir I, Omer A, Cousin P, Semenova A, Taschner M, Dergai O, Marzetta F, Iseli C, Eliaz Y, Weisz D, Shamim MS, Guex N, Aiden EL & Gambetta MC (2021) CTCF loss has limited effects on global genome architecture in *Drosophila* despite critical regulatory functions. *Nat. Commun.* **12:** 1011

Kilic S, Felekyan S, Doroshenko O, Boichenko I, Dimura M, Vardanyan H, Bryan LC, Arya G, Seidel CAM & Fierz B (2018) Single-molecule FRET reveals multiscale chromatin dynamics modulated by HP1α. *Nat. Commun.* **9:** 235

Laprell F, Finkl K & Müller J (2017) Propagation of Polycomb-repressed chromatin requires sequence-specific recruitment to DNA. *Science* **356:** 85–88

Love MI, Huber W & Anders S (2014) Moderated estimation of fold change and dispersion for RNA-seq data with DESeq2. *Genome Biol.* **15:** 550–21

Love MI, Soneson C, Hickey PF, Johnson LK, Pierce NT, Shepherd L, Morgan M & Patro R (2020) Tximeta: Reference sequence checksums for provenance identification in RNA-seq. *PLoS Comput. Biol.* **16:** e1007664

Morata G & Ripoll P (1975) Minutes: Mutants of Drosophila Autonomously Affecting Cell Division Rate. *Dev. Biol.* **42:** 211–221

Patro R, Duggal G, Love MI, Irizarry RA & Kingsford C (2017) Salmon provides fast and bias-aware quantification of transcript expression. *Nat. Methods* **14:** 417–419

Pengelly AR, Kalb R, Finkl K & Müller J (2015) Transcriptional repression by PRC1 in the absence of H2A monoubiquitylation. *Genes Dev.* **29:** 1487–1492

Scheuermann JC, De Ayala Alonso AG, Oktaba K, Ly-Hartig N, McGinty RK, Fraterman S, Wilm M, Muir TW & Müller J (2010) Histone H2A deubiquitinase activity of the Polycomb repressive complex PR-DUB. *Nature* **465:** 243–247

Thåström A, Lowary PT, Widlund HR, Cao H, Kubista M & Widom J (1999) Sequence Motifs and Free Energies of Selected Natural and Non-natural Nucleosome Positioning DNA Sequences. *J. Mol. Biol.* **288:** 213–229

Zacher B, Michel M, Schwalb B, Cramer P, Tresch A & Gagneur J (2017) Accurate Promoter and Enhancer Identification in 127 ENCODE and Roadmap Epigenomics Cell Types and Tissues by GenoSTAN. *PLoS One* **12:** e0169249
